# Revisiting the Preparation and Catalytic Performance of a Phosphine-Modified Co(II) Hydroformylation Precatalyst

**DOI:** 10.1021/jacs.4c04239

**Published:** 2024-07-02

**Authors:** David R. Holzknecht, Alexandra K. Van Alstine, Brandon P. Russell, David J. Vinyard, Fabrizio Donnarumma, Matthew B. Chambers

**Affiliations:** † Department of Chemistry, Louisiana State University, Baton Rouge, Louisiana 70803-1804, United States; ‡ Department of Biological Sciences, Louisiana State University, Baton Rouge, Louisiana 70803-1804, United States

## Abstract

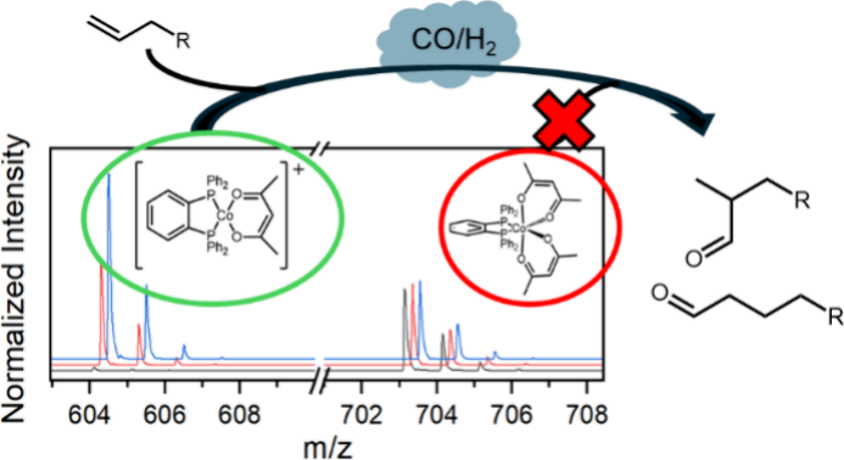

In light of recent conflicting reports regarding the hydroformylation catalytic activity derived from cationic Co(II) precatalysts of the form [Co(acac)(bis(phosphine))]BF_4_, the synthetic procedures and characterization of [Co(acac)(dppBz)]BF_4_, **1**, are evaluated. Leveraging calibrated ESI-TOF MS methodologies, substantial quantities of Co(acac)_2_(dppBz), **2**, were observed within samples of **1**. The source of the impurity, **2**, is determined to derive from incomplete protonolysis of the Co(acac)_2_ precursor and ligand scrambling occurring during the synthesis of **1**. Revised synthetic procedures using lower temperature conditions and longer reaction times afford analytically pure samples of **1** based on ESI-TOF MS and NMR spectroscopic analysis. Complex **1** is demonstrated to act as a hydroformylation precatalyst for the conversion of 1-hexene to 1-heptanal under relatively mild conditions at 51.7 bar and 140 °C. The presence of impurity **2** is shown to dramatically decrease the catalytic performance derived from **1**.

## Introduction

**Scheme 1:**
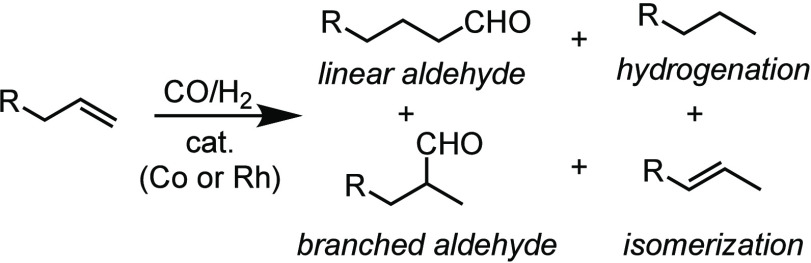
Generalized Representation of the Hydroformylation Reaction and Common Byproducts

Hydroformylation is the process in which alkenes and syngas mixtures (H_2_:CO) are directly converted into aldehyde products. It is one of the largest homogeneously catalyzed reactions, with over 10 million metric tons of aldehydes produced annually via hydroformylation (Scheme 1).^1−5^ Originally reported in 1938 by Otto Roelen, this reaction was proposed to be catalyzed by HCo(CO)_4_ generated *in situ* at elevated temperatures and pressures.^6^ While HCo(CO)_4_ remains one of the most active catalysts, it typically decomposes into Co metal at lower CO pressures, requiring the process to be run at 180 °C and over 200 bar.^7^ Co catalysts can decompose through the loss of CO at lower pressures, allowing for bimetallic species to form at the open coordination site.^8^ Through the subsequent elimination of H_2_ or other radical pathways, Co dimers and clusters are formed that ultimately lead to the decomposition to Co metal.^9−11^ If Co metal does deposit, it is capable of mediating alkene hydrogenation to alkanes, which have little commercial value and are often treated as waste.^12^ Several reports have demonstrated the viability of cobalt catalysts for hydroformylation at milder conditions (often 140 °C and 50 bar), but typically at the expense of activity, selectivity, and stability.^13−21^

Phosphine-modified catalysts were first reported in the late 1960s with proposed catalysts of the form HCo(CO)_3_(PR_3_) displaying slower rates but increased stability at lower pressures.^22^ Alternatively, in the 1970s, analogous Rh systems were reported, with HRh(CO)_4_ being the most active hydroformylation catalyst known under most conditions.^4,23−30^ Phosphine-modified Rh catalysts similarly afford stability at lower pressures.^4,30^

The advantage of phosphine modification of both the Co and Rh catalysts is that it enhances stability at lower pressures. Additionally, the presence of the phosphine ligand affords the opportunity to influence steric hindrance at the active site, which has been shown to directly impact the linear to branch ratio (*l:b*) of aldehyde products (more steric congestion favors the linear product).^4,30^ Further opportunities to modify the metal center are limited, as additional phosphine substitution increases the electron-richness of the metal center, which enhances the backbonding to the metal-bound CO substrate. This increased bond strength is the origin of the increased catalyst stability at lower pressures, but ultimately can render the M–CO moiety inert to the requisite migratory insertion and substitution steps needed for the overall catalytic transformation. Thus, despite additional phosphine modification being advantageous to stability and selectivity, current hydroformylation catalysts are limited in the extent and nature of the phosphine modification. Of note, some Rh catalysts display enhanced activity upon phosphine modification when π-accepting phosphites are incorporated.^4^

**Figure 1:**
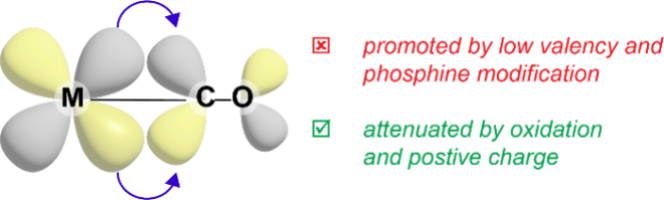
π backbonding interaction causing deleterious impacts on hydroformylation catalyst activity.

The origin of this limitation is the neutral and monovalent nature of the Co and Rh catalysts. These monovalent group IX metal centers are well-suited to back-donate to CO, with ν(MC–O) values often as low as 1950 cm^–1^, seen in CpCo(CO)_2_.^31^ Conceptually, this backbonding could be mitigated by altering the charge and oxidation state of the catalyst (Figure 1). The viability of Co(II) precatalysts for hydroformylation was initially reported by Banerjee et al., as Co(acac)_2_(H_2_O)_2_ was shown to act as a catalyst with temperatures as low as 130 °C and pressures of H_2_:CO around 100 bar for the hydroformylation of cyclohexene.^13^

Despite this report, phosphine modification of Co(II) precatalysts was not demonstrated until 2020 by Hood et al., in which a cationic Co(II) mono(acetylacetonate) platform supported by (bis)phosphine ligands was reported to generate a hydroformylation catalyst with activity approaching that of Rh(I) systems at 140 °C and 50 bar of syngas.^32^ Both the cationic nature of the precatalyst and the more oxidized state of the Co are hypothesized to attenuate backbonding to CO and to be critical in achieving the remarkable activity upon phosphine modification.^32^

Inspired by this report, Zhang et al. sought to further develop these phosphine-modified precatalysts but reported that they were unsuccessful.^33^ The authors ultimately proposed that the activity previously observed by Hood et al. was resulting from Co(acac)_2_ impurities that promoted the *in situ* generation of HCo(CO)_4_ from the Co(II) precatalyst. This was primarily supported by *in situ* IR spectroscopy studies in which features commonly ascribed to HCo(CO)_4_ were observed under hydroformylation conditions when beginning with Co(acac)_2_ as a precatalyst.^33^ When samples of [Co(acac)(dppBz)]BF_4_ and [Co(acac)(dppe)]BF_4_ (dppBz = 1,2-bis(diphenylphosphino)benzene, dppe = 1,2-bis(diphenylphosphino)ethane) prepared by Zhang et al. were used as precatalysts, no hydroformylation activity was observed.^33^ Stanley et al. responded with several *in situ* spectroscopy studies (NMR, IR, EPR) to provide evidence that the active species in their studies was not HCo(CO)_4_.^34^

In an effort to disambiguate these observations, we noted that there were significant differences in the characterization of the cationic Co(II) complexes between the two reports. There was a reliance on unassigned ^1^H NMR spectroscopy of the paramagnetic complexes and ESI-MS data that indicated the potential presence of various impurities, such as free (bis)phosphine ligands and neutral Co(acac)_2_(disphosphine) complexes.^32,33^ This was also observed by Stanley et al., but no resolution to these differences was provided.^34^ We hypothesized that the lack of a reliable characterization protocol was limiting the assessment of these complexes as precatalysts for hydroformylation and the root cause of the differences in catalytic performance.

Herein, we evaluate the reproducibility of [Co(acac)(dppBz)]BF_4_ as a hydroformylation precatalyst following prior synthetic protocols. We correlate variances in activity to features present in ESI-MS data. Leveraging calibrated ESI-MS techniques, we optimize the synthetic protocol for [Co(acac)(dppBz]BF_4_ and demonstrate a highly reproducible method for observing hydroformylation catalysis. While not an absolute determination of sample purity due to differences in ionization capacity and fragmentation patterns, ESI-MS provides keen insights into relative sample composition with appropriate response calibrations for critical species of interest.

## Results and Discussion

### Initial Catalysis Evaluation Using Literature Protocols

Following the observations by Zhang et al., replicating the hydroformylation activity of [Co(acac)(dppBz)]BF_4_ (**1**) proved to be initially difficult in our own hands (Table 1). In over 30 catalytic assays encompassing dozens of batches of **1**, we observed hydroformylation activity for the conversion of 1-hexene to heptanal at 51.7 bar of syngas and 140 °C. However, the catalytic activity of **1** was inconsistent (Table 1). The average aldehyde yield in our studies was only 5% lower than what was published by Hood et al., but the standard deviation of the measurement is ±11%.^32^ The extremes of the performance emphasizes this issue in aldehyde yield (highest conversion 65%, lowest 24%).

**Table 1:**
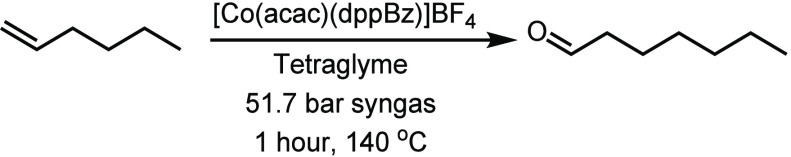
Hydroformylation of 1-Hexene by [Co(acac)(dppBz)]BF_4_^a^

	Aldehydes (%)	Isohexenes (%)
Hood et al.^32^	52	14
Highest^b^	65	16
Lowest^b^	24	12
Average^b^	47 ± 11	19 ± 6

a Reaction conditions: 1 mM **1**, 1 M (11.2 mL) 1-hexene, 78.8 mL of tetraglyme, 51.7 bar of syngas, 140 °C, 1 h.

b This work.

To explain the variability in hydroformylation activity, we hypothesized that the purity of the precatalyst across synthetic batches was not consistent. The primary method of characterization from the initial report was ^1^H NMR spectroscopy of **1**, which we replicated qualitatively (Figure S1). Broad features diagnostic of paramagnetic species are seen between 31.7 and −7.4 ppm in CD_3_CN with other sharper resonances in a diamagnetic range between 8.0 and 2.1 ppm. Notably, the ratio of the paramagnetic features to the resonances within the diamagnetic range was found to vary from batch to batch of **1** and were not replicated at all in the report by Zhang et al. claiming to prepare the identical complex. When compared across several independently prepared batches of **1**, we were unable to directly correlate activity to spectral features in the ^1^H NMR spectra (Figure S2). Given this difficulty in assigning and interpreting the paramagnetic ^1^H NMR data, a greater focus was placed on mass spectrometry as a characterization method for **1**.

Complex **1** was previously characterized by ESI-TOF MS using two different but analogous methods, but such data were not able to be replicated within our laboratory. Previous solvent conditions for ESI-TOF MS of **1** reported by Zhang et al. and Hood et al. utilized an aqueous formic acid cosolvent with either MeOH or acetonitrile (ACN). When replicating these conditions, upon dissolution we observed instantaneous formation of a white solid. Upon isolation and characterization by ESI-TOF MS, the white solid was found to have a molecular ion peak of 447.1433 *m*/*z*, which correlates closely to protonated dppBz (theoretical *m*/*z* = 447.1431, Δ*m* = 0.45 ppm) (Figure S13). Simultaneously, in the spectra shown in Figure S12A and S12B, the desired 604.1131 *m*/*z* signal peak corresponding to [Co(acac)(dppBz]^+^ is observed to be barely distinguishable from the baseline of the spectra. Thus, we observed that the material capable of catalyzing hydroformylation was not stable in the presence of formic acid, and we sought an alternative mass spectrometric protocol.

### Redesigning the Mass Spectrometry Characterization Methodology

**Figure 2:**
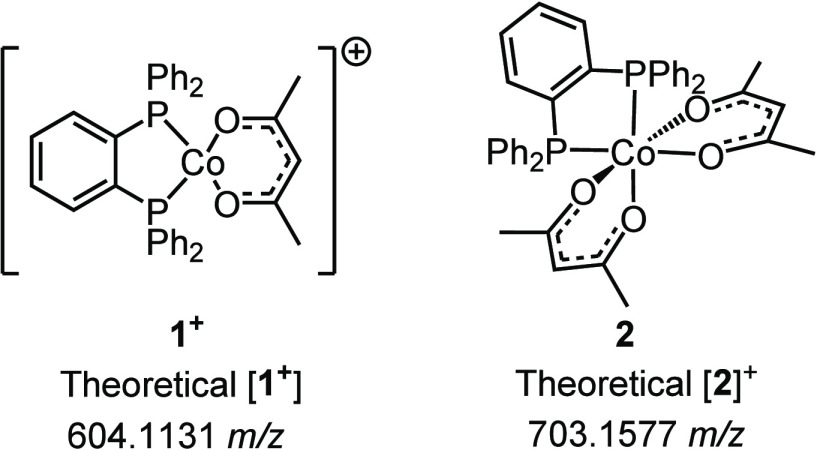
Primary components observed in ESI-TOF MS data.

**Figure 3:**
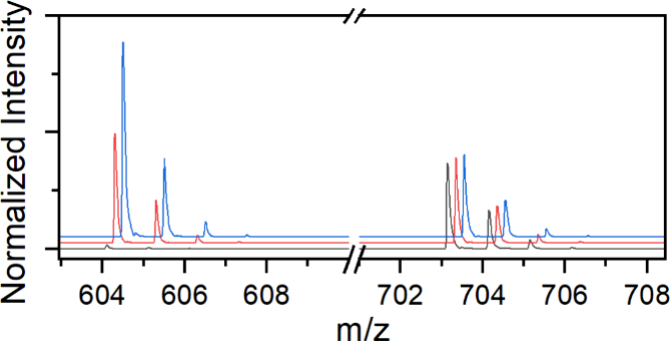
Mass spectra of three representative batches of **1** in acetonitrile, normalized to the intensity of 703.1560 *m*/*z*, corresponding to the presence of **2**. An *xy* offset was applied to the spectra for visual clarity.

The ESI-TOF MS protocol used throughout the rest of the studies is detailed fully in the Experimental Methods section (*vide infra*). Data were initially collected in ACN in the absence of the formic acid/water cosolvent. The ESI-TOF MS data of **1** in ACN exhibited several features, but the two with the highest intensity possess one charge unit and are assigned to [Co(acac)(dppBz)]^+^ (found 604.1120 *m*/*z*, Δ*m* 10 ppm, theoretical 604.1131 *m*/*z*) and [Co(acac)_2_(dppBz)]^+^ (found 703.1560 *m*/*z*, Δ*m* 10 ppm, theoretical 703.1577 *m*/*z*) and depicted in Figure 2. Figure 3 shows a representative set of three ESI-TOF MS spectra collected in the absence of acid.

Each spectrum is taken from an independently prepared batch of **1** and normalized to the major impurity at 703.1560 *m*/*z*. Of note, these spectra consistently show less free dppBz (447.1431 *m*/*z* for [dppBzH]^+^). These observations support the hypothesis that acidic conditions lead to decomposition of **1** via protonolysis of the ligand environment by formic acid. Additionally, the absence of acid results in fewer features at higher *m*/*z* values (>900) assigned to cobalt oxide cluster impurities, possibly the outcome of acid-induced decomposition. Like the catalytic activity observations, the reproducibility in this measurement was low. The relative intensity of the desired product [Co(acac)(dppBz)]^+^ ranged from being the largest peak (Figure 3, blue trace) in the spectrum to practically nonexistent (Figure 3, black trace).

While the use of acid-free ACN eliminated the presence of free dppBz in the ESI-TOF MS data, a high degree of variability in the relative intensity of 604.1120 *m*/*z* (assigned to [Co(acac)(dppBz)]^+^) relative to other signals was still observed.

The feature at 703.1560 *m*/*z*, assigned to [Co(acac)_2_(dppBz)]^+^, is derived from Co(acac)_2_(dppBz) (**2**) impurities remaining from the initial synthesis of **1**. We noted the possibility of Co(acac)_2_(dppBz) fragmenting during the analysis to afford the signals assigned to **1**. To evaluate this, bis(acac) complex (**2**) was independently prepared through the addition of dppBz to Co(acac)_2_. The resulting ESI-TOF mass spectrometry data for **2** was collected (Figure S7), and no evidence for the formation of [Co(acac)(dppBz)]^+^ (604.1220 *m*/*z*) was observed. This supports the assertion that the feature at 604.1220 *m*/*z* originates from the unpairing of the counterion, BF_4_^–^, from **1** and not fragmentation of **2**. This also allows for the assignment of **2** as an appreciable impurity in many samples of **1**.

**Figure 4:**
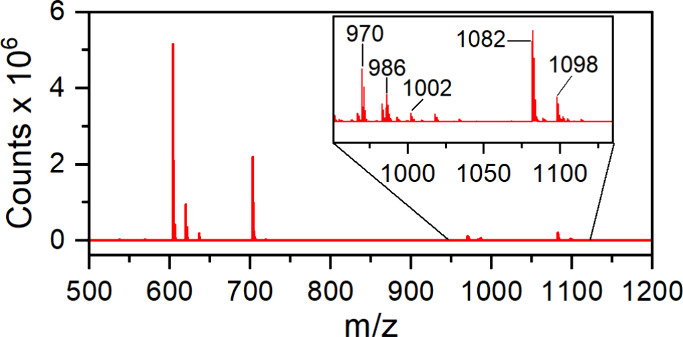
ESI-TOF mass spectrum of **1** in acetonitrile with an inset figure enhancing the 950–1150 *m*/*z* region.

Beyond the presence of **2** as an impurity in the synthesis of **1**, there are several features at larger *m*/*z* values. The features at *m*/*z* > 900 are highly inconsistent between samples, but those that are consistently observed are correlated to additional peaks separated by 16 *m*/*z* units (e.g., 970 *m*/*z*, 986 *m*/*z*, and 1002 *m*/*z*; 1082 *m*/*z* and 1098 *m*/*z* in Figure 4). We hypothesized that these features are derivative of Co-oxides with sequential addition of oxygen that result from decomposition of **1** (notably proposed to be a 15-electron species with open coordination sites) and **2** in solution. Since the only sources of oxygen atoms in solution would be from either acac or adventitious H_2_O or O_2_ during ESI-TOF MS sample preparation, we suspected that exposure to ambient air was deleterious. Even when considering the features attributed to **1** (604.1120 *m*/*z*), peaks were observed at 620.1210 *m*/*z* and 636.1074 *m*/*z*, which correlates to the presence of the features at *m*/*z* > 900.

**Figure 5:**
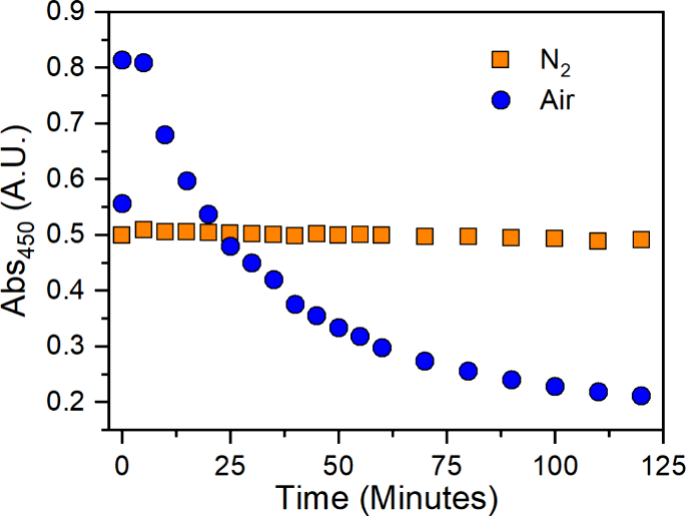
Absorbance profile of a 0.5 mM solution of **1** in ACN monitoring 450 nm over time under N_2_ and after air exposure.

To explore the possible sensitivity of solutions of **1** to ambient atmospheric conditions, we analyzed the stability of **1** in acetonitrile over time by UV–vis spectrophotometry (Figures S14 and S15). Using the absorptivity at 450 nm as a guidepost (Figure 5), under N_2_, minimal variation of the UV–vis spectrum is observed over 2 h, indicating substantial solution stability. A separate but identical solution of **1** was exposed to air after taking an initial spectrum under N_2_. Upon air exposure, the 450 nm absorption sharply increased in its absorbance by nearly 50% (Figure 5) within 5 min. The absorbance then exponentially decays over the next 2 h. From the maximum absorption, the exponential decay has an estimated half-life of about 30 min in ACN (Figure 5). Furthermore, the significant increase in absorbance at 450 nm immediately after air exposure suggests an initial rapid reaction with O_2_ and/or H_2_O. Thus, exposure of solutions of **1** to ambient air prior to ESI-TOF MS analysis stands to influence sample composition.

The rapid decomposition of **1** in ACN when exposed to air led to the revision of our MS sample preparation procedure. Previously, solid samples were removed from an N_2_-filled glovebox in a screw-cap vial and brought to the mass spectrometer. The solutions for analysis were prepared in an adjacent fume hood and transferred to the instrument over the course of an estimated 20 min (Figure S16a).

To prevent air-assisted decomposition of **1**, solutions for MS analysis were directly prepared in an N_2_-filled glovebox and placed in Teflon-capped autosampler vials with 300 μL conical inserts. These vials were wrapped with Para Film, placed within a 20 mL scintillation vial, and closed prior to removal from the glovebox. Upon removal, samples were transferred to the mass spectrometer for analysis immediately upon removal of the vial cap. This approach reduced the exposure time of the sample inside the vial to a few seconds. For quantification of sample purity, all samples were taken in triplicate to assess the error of the measurement. Triplicate data could not be replicated from one singular solution of **1** since piercing the septum of the sample vial within the spectrometer is still observed to afford air-assisted decomposition of the complex over time (Figure S16b). To collect triplicate data on a batch of **1**, three individual samples were made from one batch of material.

**Figure 6:**
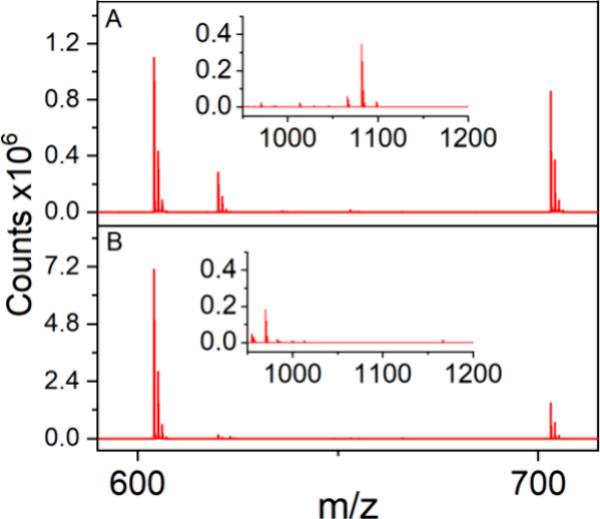
Positive ionization mode mass spectra of **1** in CH_3_CN. (A) Data acquired using previously reported MS protocol.^32^ (B) Data acquired employing anaerobic protocol presented within this work.

To assess the sample preparation procedure, we compared the relative intensity of the ESI-TOF MS signal of **1** to the signal for complex **2** impurity between samples prepared under ambient conditions to those prepared within an N_2_-filled glovebox. The samples prepared in open air were found to have a 14% relative reduction in the feature assigned to **1** when identical batches were analyzed (Figure S17). In addition, the diagnostic oxygen contamination peak at 620 *m*/*z* is reduced by 75%, and the >950 *m*/*z* region exhibits substantially less contamination by relative intensity (Figure 6). Adding to the improved signal for the feature assigned to **1** and fewer oxygen contamination peaks, this method also yielded consistent purity measurements for **1** with errors as low as ±1% (Table S17).

### Revised Synthesis and Characterization of Complex **1**

**Scheme 2:**
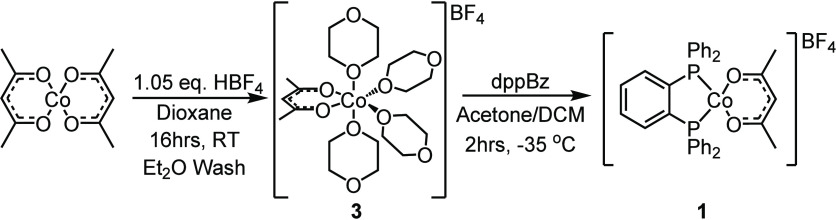
Modified Synthesis of [Co(acac)(dioxane)_4_]BF_4_ and [Co(acac)(dppBz)]BF_4_

With the determination of a reliable characterization method, we turned our attention to optimizing the synthesis of **1** to minimize the amount of the complex **2** impurity. To identify the source of **2**, the precursor to **1**, [Co(acac)(dioxane)_4_]BF_4_ (**3**), was assessed by ESI-TOF MS. Precursor **3** is prepared upon treatment of commercially available Co(acac)_2_ with 1 equiv of HBF_4_ in Et_2_O. The ESI-TOF MS analysis of **3** indicated that Co(acac)_2_ (at 257.0220 *m*/*z* for [Co(acac)_2_]^+^) remains in the sample postprotonolysis (Figure S9). Excess Co(acac)_2_ carrying over to the dppBz addition step is likely a source of some of impurity **2** within samples of **1**. We determined that **3** can be prepared in greater yields by addition of HBF_4_ under N_2_ at room temperature in a dioxane solution (Scheme 2). The reaction is allowed to stir for 16 h followed by vacuum filtration to isolate the precipitate **3**. The pink solid is then washed with Et_2_O to remove any excess HBF_4_. Under these conditions, ESI-TOF MS analysis indicated no evidence for residual Co(acac)_2_ (Figure S11). The primary difference of the modified synthetic procedure is that the HBF_4_ was not added under elevated temperatures in contrast to what was previously reported.^32^

Independent batches of **1** were synthesized using precursor **3** prepared from the revised procedure (**3**^**rev**^) and compared to batches synthesized from the original higher temperature procedure (**3**) by ESI-TOF MS in triplicate. The modified precursor **3**^**rev**^ resulted in a purer sample of **1**, as evidenced by the ESI-TOF MS data (Figure S18). Using an uncalibrated peak area analysis, the sample composition is determined to be enriched in complex **1**, but only by 7% when prepared to **3**^**rev**^ (81% vs 74% purity of **1** with respect to **2**). This indicates that the Co(acac)_2_ present in the initial precursor **3** likely contributes to the presence of bis(acac) impurity **2** but is not the sole source of the impurity. We thus conclude that while an impurity of **2** can form from residual Co(acac)_2_ contamination, an alternative pathway is also relevant.

**Figure 7:**
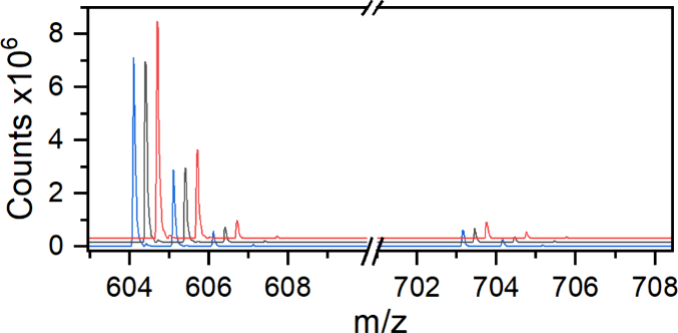
Mass spectra of one batch of **1** in triplicate. ESI-TOF analysis of **1** synthesized at −35 °C in triplicate. The spectra were offset and zoomed into the 604.1120 *m*/*z* and 703.1560 *m*/*z* regions for visual clarity.

As Co(II) is well-known for its ability to undergo ligand substitutions,^35−37^ the mono(acac) precursor [Co(acac)(dioxane)_4_]BF_4_ could undergo ligand rearrangements prior to coordination of dppBz to generate impurity **2**. To inhibit the possible ligand scrambling to yield **2** during the synthesis of **1**, reactions were run at −35 °C and allowed to proceed for 2 h (Scheme 2). The lower temperature is hypothesized to disfavor competing ligand scrambling kinetically. Samples of **1** prepared using the low-temperature synthetic method were analyzed by ESI-TOF MS across multiple batches, and the relative intensity of the signal assigned to **2** was reduced by 80% relative to the original synthetic method (Figure 7). The uncalibrated peak area analyses for 604.1120 *m*/*z* (**1**) and 703.1560 *m*/*z* (**2**) are shown in Table S18. The purity of **1** shows little variability across batches. Peaks in the MS data associated with free ligand, air contamination, and other decompositions/ligand rearrangements are seen as a trace with a relative peak intensity of <1% (Figure S4).

Thus, the lower temperature synthesis for the conversion of **3**^**rev**^ to **1** led to significant enhancements in purity of the desired [Co(acac)(dppBz)]BF_4_ complex (Scheme 2). Taken together, our observations indicate that thermal ligand scrambling from precursor **3** and **3**^**rev**^ is the primary source of a bis(acac) coordination environment that ultimately leads to impurity **2**. Upon formation of **1**, no additional ligand scrambling to a bis(acac) complex has been observed.

With ligand scrambling occurring during the preparation of **1**, we explored the possibility of ligand scrambling when the precatalyst is activated. True post-mortem analysis of reaction mixtures is hindered due to using tetraglyme as the solvent, so we heated a 1.3 mM solution of **1** in toluene at reflux for 30 min. An N_2_ atmosphere was used instead of H_2_/CO to eliminate any activation and directly interrogate ligand scrambling and decomposition pathways. ESI-TOF MS analysis of the samples postheating indicates **1** does decompose (Figure S22). Only 11% of the decomposition yields **2** (Table S25), with the remaining going toward unidentified products, many of which give signals in the 900–1050 *m*/*z* range. Similar thermolysis studies of **2** resulted in no evidence of decomposition under those conditions (Figure S23). Since **2** appears stable, the changes in the ESI-TOF MS data upon heating **1** are attributed to the decomposition of **1** as opposed to an intermediate conversion to **2** prior to decomposition. We conclude that, while ligand scrambling is possible when activating **1**, this is a minor pathway and nonspecific thermal decomposition would be more competitive to the activation process.

With an optimized ESI-TOF MS procedure and a revised synthesis, all batches of material were analyzed in triplicate to further assess reproducibility. Figure 7 demonstrates the increased purity and reproducibility upon changing the method for MS sample preparation, the synthesis of **3**^**rev**^, and the synthesis of **1**. The purity based on an uncalibrated analysis of peak areas of the features observed in the ESI-TOF MS data was determined to be 94 ± 1%. This contrasts with the previously reported synthetic procedure that never exceeded 80% for **1** in our hands. Additionally, the deviation of the amount of **1** observed when a single batch was analyzed multiple times was 1%, which gives confidence in quantifying the purity of **1** in a reproducible manner.

With the ability to independently prepare **1** and **2**, we sought to calibrate the ESI-TOF MS response factors for the two species to quantify better the purity of the material being prepared. Two calibration curves for the ESI-TOF MS instrument were created in which samples of **1** and **2** were independently prepared at various concentrations (1, 5, 10, 25, and 50 μg/mL) (Figures S20 and S21). The response factor of **1** is found to be 6.5 × 10^5^ counts/(μg/mL), while the response factor for **2** is 8.7 × 10^5^ counts/(μg/mL). The larger response factor for **2** indicates that the amount of impurity **2** was overestimated in the uncalibrated peak area analysis. The neutral charge of **2** and additional chelating acac ligand potentially allow for more facile ionization of **2** to nominally yield a Co(III) state within the mass spectrometer.

Using the same LC-MS data from Figure 7 for Table S18, the purity of **1** can be reassessed, accounting for the response factors of the two complexes. This results in a 7% increase in the purity of the samples of **1** from 93% to >99%. Thus, the revised characterization methods and synthetic procedures allow for the reproducible preparation of [Co(acac)(dppBz]BF_4_ in near 99% purity. Representative ESI-TOF MS data for three independently prepared batches of **1** are shown in Figure S19.

Of note, regardless of synthetic protocol or purification efforts, ^1^H NMR spectroscopy continually indicated that dioxane was present in all samples of **1**. This suggests that 1,4-dioxane present in [Co(acac)(dioxane)_4_]BF_4_ is retained within the sample of **1** during the synthesis. The lone reported crystal structure analogous to **1** bears an apically bound THF molecule and has the formula [Co(acac)(dppBz)(THF)]BF_4_.^32^ While initially considered to be an artifact of crystal packing, we hypothesized that the fifth ligand could be a required feature of the structure of **1** and that dioxane could be present as a fifth ligand. Electronically, a four-coordinate Co(II) complex would formally have 15e^–^, so the inclusion of a fifth ligand seems highly likely. In an effort to remove the 1,4-dioxane from **1**, a sample was dried under vacuum over a 5-day period. Then 13.6 mM TMS was added to a sample of **1** dissolved in CD_3_CN as a ^1^H NMR spectroscopy standard (Figure S5). Based on the integrations of TMS and the dioxane peak, we determined that there is approximately 1.5 equiv of 1,4-dioxane per cobalt center. While efforts to crystallize **1** have been unsuccessful, the presence of a fifth ligand seems highly likely and should be considered as part of the primary coordination sphere of **1**.

With confidence in the synthetic and characterization protocols for both **1** and **2**, we explored the possibility of using EPR spectroscopy to evaluate sample composition. The EPR spectra for the complexes were distinct. The spectrum of **1** in toluene was collected at 77 K and agreed with the previously reported data collected at 5.5 K.^32^ A *g*_iso_ value of 2.30 was measured, but unlike the data collected at 5.5 K, no hyperfine coupling to the ^59^Co nuclei was observed (Figure S24). The EPR spectra at 77 K for **2** in toluene afforded an axial signal, *g* = [2.31, 2.02, 2.00], with additional high field splitting from the ^59^Co with an estimated hyperfine constant of 25 gauss (Figure S25). So, while EPR spectroscopy at 77 K can be used to identify the presence of **2**, it would be insufficient to characterize **1**, given that the only observable parameter is the *g*_iso_ value of 2.30. Spectra collected at 5.5 K would allow for a more conclusive characterization of **1** with the observable diagnostic hyperfine interaction between the ^59^Co and the ^31^P nuclei.

### Hydroformylation Activity with Complex **1** as Precatalyst

With confidence in the purity of **1** synthesized via the revised synthetic method, we reassessed **1** as a precatalyst for hydroformylation. Catalytic assays of three different batches of **1** were each tested in triplicate for the hydroformylation of 1-hexene (Table 2). Following similar procedures reported by Hood et al. and Zhang et al., 1 mM solutions of **1** in tetraglyme solvent samples were heated to 140 °C and pressurized to 51.7 bar of syngas in the presence of 1 M 1-hexene for 1 h. Products of hydroformylation were quantified by GC-MS analysis.

**Table 2:** Hydroformylation of 1-Hexene by **1** in Tetraglyme^a^

	Total TONs	Total Product Yield (%)	Total Aldehyde Yield (%)	*l:b*	Isohexenes Yield (%)
Hood et al. Protocol^b^	801	66	47	0.95	19
Std. Dev.	158	13	11	0.1	6
Revised Protocol Average	850	70	53	1.1	17
Revised Protocol Highest	935	75	58	1.1	17
Revised Protocol Lowest	776	64	49	1.0	15
Std. Dev.	49	3	3	<0.1	1

a 1 M 1-hexene (11.2 mL), 0.82 mM 1, and 78.7 mL of tetraglyme.

b Precatalyst **1** prepared following methods reported in ref (32).

For samples of **1** prepared by our revised synthetic method, the average yield was determined to be 70%, representing 850 turnovers. This is an increase from the average observed yield of 66% and 800 turnovers from samples prepared as previously described. For both the materials, isomerization of 1-hexene accounted for about 14–17% of the product with the rest being aldehydes.

Illustrative of the more reliable catalytic performance resulting from samples of **1** prepared and characterized through our revised methods, the minimum TON for this purer version of **1** was greater than the average TON observed using samples prepared from the Hood et al. and Zhang et al. methods. The linear to branched aldehyde product ratio (*l:b*) of the new synthetic method material is slightly increased from 0.95 to 1.1, but remains close to 1.

Importantly, the reproducibility of the activity of **1** prepared via the revised protocol is now greatly improved. In agreement with the increased consistency of the mass spectrometry data, the reproducibility of the catalytic data is much greater than the results shown in Table 1, which uses the previous methods for preparing **1**. The elimination of **2** from the sample greatly reduced the error in our measurements and could explain the discrepancy in the data sets of Hood et al. and Zhang et al. As the purity of the sample increased, so did its activity and selectivity for the linear aldehyde.

**Figure 8:**
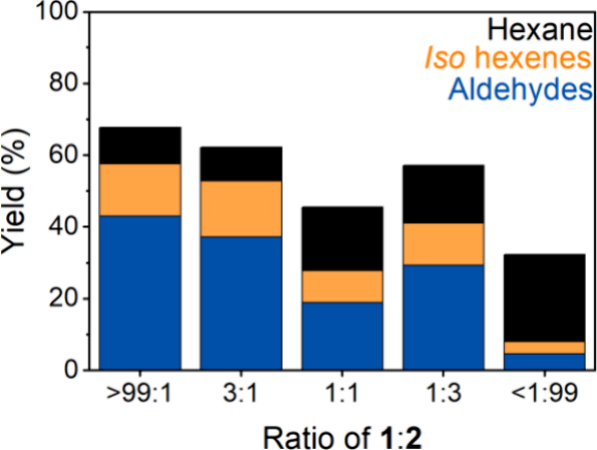
Reaction product distribution versus the ratio of 1:2. (Black) hydrogenation, (orange) isomerization, (blue) hydroformylation. 1 M 1-hexene (11.2 mL), 1 mM combined precatalyst (1 and 2), 0.1 M heptane (1.3 mL) as a GC-MS standard, and 77.5 mL of tetraglyme.

With the expectation that the activity of **1** is hindered by the presence of **2**, we explored various rationally prepared precatalyst loadings of **1** and **2** in a precatalyst mixture to quantify this effect. Ratios of **1**:**2** of 3:1, 1:1, and 1:3 were assessed for catalytic performance while maintaining a constant cobalt concentration of 1 mM. The data for each precatalyst ratio were collected in triplicate, and the full data set can be seen with error analysis in Table S20. For this data set, 0.1 M heptane was included as an internal standard for the GC-MS analysis to determine the manner in which **2** influences the production of hexane generated via hydrogenation of 1-hexene. The summation of the various product yields is shown in Figure 8. The >99:1 data point showed an overall yield in line with our average in Table 2 at 70%.

In contrast to the report by Hood et al., the pure complex **1** produced a substantial amount of hexane (18% yield). Of note, hexane is the sole product analyzed indirectly and quantified through mass balancing the observed species in the catalytic mixture (see SI pg. 15). Independent calibration of the GC-MS response factors of observed aldehydes and internal alkenes allows for the assignment of substantial hexane formation during the hydroformylation of 1-hexene.

Independently prepared samples of **2** were also studied as precatalysts, which is represented by the <1:99 data point. **2** demonstrated minimal activity toward the hydroformylation of 1-hexene and preferentially hydrogenates 1-hexene to hexane. It is of note that the total activity is substantially lower than **1** (28% of 1-hexene consumed vs 70% 1-hexene consumed). This represents not only a dramatic change in the overall catalysis but a significant decrease in the rate of substrate consumption when **2** in present.

As more **2** is added to assays containing **1**, a general decrease in activity is observed. However, the 1:1 ratio (50% **1** and 50% **2**) is substantially less active than the 1:3 (25% **1** and 75% **2**) precatalyst ratio. This indicates that there is an influence of **2** on the activation of **1** that results in the nonlinear dependence of [**2**] on catalytic performance. Nevertheless, the overall trend of the data points shows the presence of increasing amounts of **2** hinders catalysis and that certain combinations of **1** and **2** could lead to significantly less catalytic activity than expected.

**Figure 9:**
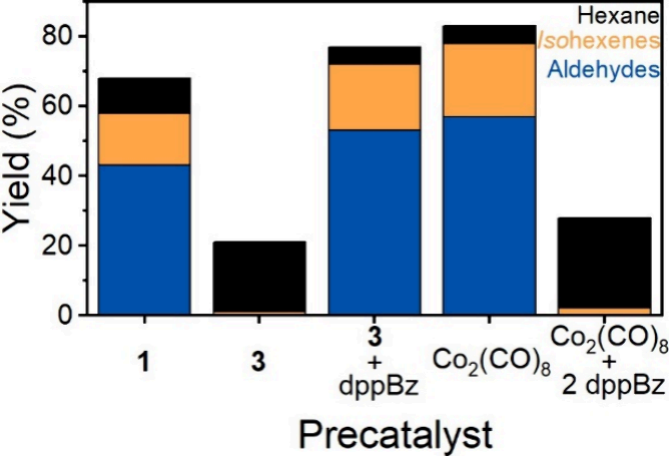
Reaction product distribution derived from various precatalyst mixtures under standard catalytic conditions: 0.1 M heptane (1.3 mL) as a GC-MS standard and 77.5 mL of tetraglyme. Total Co concentration was maintained at 1 mM. (Black) hydrogenation, (orange) isomerization, (blue) hydroformylation.

These observations align with traditional Co(I) hydroformylation chemistry in which the presence of multiple phosphine ligands slows the hydroformylation process and promotes hydrogenation.^4,17,22,38,39^ This also agrees with reports of neutral Co(II) phosphine complexes acting as efficient hydrogenation catalysts.^40−43^ As has been previously postulated,^33^**1** may be an avenue to an active HCo(CO)_4_ catalytic state. To evaluate this, we measured the hydroformylation activity of the Co_2_(CO)_8_ precatalyst, known to generate the canonical HCo(CO)_4_ upon activation, in the absence and presence of dppBz (Figure 9). In the absence of dppBz, as has been shown by Zhang et al.,^33^ Co_2_(CO)_8_ is activated and mediates the hydroformylation of 1-hexene at a comparable rate to **1** at similarly mild conditions. Upon addition of a stoichiometric amount of dppBz, the catalysis for hydroformylation is inhibited completely and only hydrogenation is observed. When **3**, the unmodified precursor to **1** ([Co(acac)(dioxane)_4_]BF_4_), is used as a precatalyst, negligible hydroformylation is observed and alkene hydrogenation dominates reactivity. This correlates to the formation of cobalt metal as a decomposition product within the reactor. In contrast to Co_2_(CO)_8_, when **3** is assayed in the presence of a stoichiometric amount of dppBz (Figure 9), the hydroformylation activity is similar to **1**. This difference in behavior in the presence of dppBz not only demonstrates that various phosphine-modified Co(II) precatalysts can be prepared *in situ* via the treatment of **3** with phosphines but also suggests that the catalytic activity does not derive from a traditional monometallic Co(I) hydrido carbonyl complex. While further experimental studies are required to unambiguously assign the oxidation state and structure of the active catalyst, these results show that the catalyst generated from **1** is likely unique and warrants additional interrogation.

## Conclusions

The viability of cationic cobalt(II) complexes as unique precatalysts for hydroformylation has been reassessed using [Co(acac)(dppBz]BF_4_, **1**, as an exemplary complex. In agreement with prior studies,^32,33^ activity was observed but with significant uncertainties to the measurements and minimal product formation often found. Upon redesigning mass spectrometric protocols, Co(acac)_2_(dppBz), **2**, was found as a common impurity in samples of **1** and was demonstrated to have deleterious effects on catalytic performance. With a reliable method of characterization in hand, a synthetic optimization of precatalyst **1** was performed in which lower temperatures and longer reaction times afforded samples of **1** reproducibly with negligible amounts of **2**. Hydroformylation studies subsequently demonstrate that **1** reliably generates an active catalyst with performance slightly exceeding that which was previously reported with additional hydrogenation activity identified.^32^ Furthermore, comparisons to Co_2_(CO)_8_ as a precatalyst, known to generate an active HCo(CO)_4_ catalyst that is deactivated in the presence of dppBz, indicates that the catalytic state derived from **1** is unique and unlikely HCo(CO)_4_. The further characterization of this catalytic state is ongoing. With these revised synthetic and characterization protocols reported, we aim to facilitate and encourage the further development of cationic Co(II) precatalysts for hydroformylation and other applications.

## Experimental Methods

### General Considerations

All synthetic procedures were carried out using air-free Schlenk line techniques or utilized an N_2_-filled glovebox unless otherwise stated. All chemicals were used as received unless stated otherwise. Et_2_O was purchased from VWR, degassed with argon, and purified through a Pure Process Technology solvent purification system. The Et_2_O was then transferred to the N_2_-filled glovebox, where it was stored over 4 Å molecular sieves. All other solvents and 1-hexene were purchased from commercial vendors (Honeywell, VWR, J.T. Baker, Acros Organics, Sigma-Aldrich, BTC, and Thermo Scientific), degassed with N_2_, and stored over 4 Å molecular sieves in the N_2_-filled glovebox. Co(acac)_2_ was purchased from Alfa Aesar, while Co_2_(CO)_8_ and dppBz were purchased from Strem Chemicals and stored in an N_2_-filled glovebox. CD_3_CN and D_2_O were purchased from Cambridge Isotope Laboratories and stored in an N_2_-filled glovebox following degassing by three cycles of freeze–pump–thawing for CD_3_CN and sparging with N_2_ for D_2_O.

A 400 MHz Bruker spectrometer was used to collect ^1^H NMR spectra at 25 °C. The residual solvent peak was used to reference the chemical shifts. The TopSpin 3.6.3 software suite was used to process ^1^H NMR data. UV–vis spectra were collected on an Ocean-FX-XR1-ES spectrometer from Ocean Optics with a DH-2000-BAL deuterium/tungsten source, which is controlled by the OceanView software.

High-pressure catalytic assays were performed using a 160 mL Parr reactor, which is connected to a Parr 4871 controller. The Parr 4871 controller is operated by a Windows PC using SpecView v2.5 software to process the stirring rate, pressure, and temperature of the system. Catalytic assays were analyzed by an Agilent GC-MS 6890 N Agilent Technologies GC equipped with an HP-5 ms 5% phenyl methyl silica column 30 m and 0.25 μm film attached to a 5975 B Agilent Technologies mass spectrometer. EPR spectra were recorded on a Bruker EMX spectrometer equipped with a standard ER4102 resonator. EPR spectra were measured at 77 K with liquid nitrogen “finger dewar” installed in the resonator. Acquisition parameters were frequency, 9.43 GHz; modulation amplitude, 10 G; modulation frequency, 100 kHz; time constant, 81.92 ms; conversion time, 30 ms.

### Preparation of [Co(acac)(dppBz)]BF_4_, **1**

*Method A*. The following procedure was adapted from Hood et al.^32^ Within an N_2_-filled glovebox, 0.200 g (0.32 mmol) of **3** was added to a 20 mL scintillation vial and dissolved in 5 mL of acetone. One equivalent of dppBz (0.142 g, 0.32 mmol) was added to a 20 mL scintillation vial and dissolved in 5 mL of DCM. The solution of dppBz was added to the solution containing **3** while stirring with a magnetic stir bar. The resulting dark red mixture was stirred for 30 min, after which the solution was dried under vacuum and used without further purification, affording 0.324 g of **1** as a dark red powder. Yield: >99%. ^1^H NMR (400 MHz, CD_3_CN, 24 °C) δ = 32.25 (br), 27.02 (br), 9.53 (br), 8.72 (br), 8.0–7.2 (m, br), 5.48 (s, br), 5.09 (s, br), 3.63 (s, dioxane), 2.04 (s), −7.43 ppm (br).

*Method B*. For an alternative route to **1**, 5 mL of DCM and acetone were each added separately to 20 mL scintillation vials and cooled to −35 °C in an N_2_-filled glovebox. The acetone was transferred to another scintillation vial containing 0.277 g (0.44 mmol) of **3**. The cooled DCM was transferred to a separate scintillation vial containing 0.197 g (0.44 mmol) of dppBz. While cooled, the solution of **3** in acetone was added dropwise to the vial containing dppBz in DCM. The reaction mixture was stirred well using a magnetic stir bar for 1 min and then cooled to −35 °C for 2 h. The resulting dark red solution was placed under vacuum to dry to afford 0.376 g of **1**. Yield: >99%. ^1^H NMR (400 MHz, CD_3_CN, 24 °C) δ = 31.88 (br), 9.49 (br), 8.71 (br), 8.0–7.2 (m, br), 5.47 (s, br), 5.08 (s, br), 3.63 (s, dioxane), 2.11 (s), −7.23 ppm (br).

### Preparation of [Co(acac)(dioxane)_4_]BF_4_, **3**

*Method A*. The following procedure was adapted from Hood et al.^32^ In an N_2_-filled glovebox, 0.400 g (1.56 mmol) of Co(acac)_2_ was transferred to a 25 mL 3-neck round-bottom flask containing 4 mL of 1,4-dioxane and a magnetic stir bar. The solid in the resulting suspension was observed to transition from a dark purple solid to light pink and remained mostly undissolved. 0.222 mL (0.264 g, 1.63 mmol) of HBF_4_·Et_2_O was separately added to an addition funnel and diluted with 4 mL of 1,4-dioxane. The addition funnel and 3-neck round-bottom flask were then sealed with septa and removed from the glovebox. The 3-neck round-bottom flask was connected to a Schlenk line with an N_2_-line adaptor and a condenser. While flowing N_2_ through the flask, the previously prepared addition funnel was also attached to the reaction vessel. The mostly undissolved Co(acac)_2_ suspension was heated to 60 °C while being magnetically stirred. Once all the solid dissolved at 60 °C, the solution was cooled to 40 °C, at which point the stopcock of the addition funnel was fully opened to quickly add the HBF_4_·solution to the Co(acac)_2_. Upon addition of HBF_4_, the heating was immediately stopped and the reaction mixture was allowed to return to room temperature to stir for 16 h under N_2_. Upon cooling, a light pink solid precipitated. After 16 h, the mixture was filtered in ambient air through a glass frit to isolate the light pink solid. The solid was washed with excess Et_2_O to remove any residual HBF_4_·Et_2_O. The solid was placed into a 20 mL scintillation vial and transferred into an N_2_-filled glovebox. The solid was further dried under vacuum in the glovebox to afford 0.421 g of **3** used without further purification. Yield: 55%. The 1,4-dioxane content was quantified through a destructive NMR experiment in which dissolution of **3** in D_2_O affords liberation of the 1,4-dioxane. The amount of 1,4-dioxane was quantified via the inclusion of an acetone internal standard (Figure S8): (400 MHz, D_2_O, 24 °C) δ = 3.70 (s, dioxane), 2.23 (s), 2.18 (s, acetone).

*Method B*. In an N_2_-filled glovebox, 0.400 g (1.56 mmol) of Co(acac)_2_ was transferred to a 20 mL scintillation vial containing 10 mL of 1,4-dioxane to afford a partially dissolved dark purple suspension. Then 0.222 mL (0.264 g, 1.63 mmol) of HBF_4_·Et_2_O was directly added to the stirred Co(acac)_2_ solution. The addition of HBF_4_·Et_2_O resulted in the residual purple solid fully dissolving into the solution. The solution was allowed to stir at room temperature for 16 h. During this time, a pink solid precipitated. The pink solid was filtered with a glass frit and washed with a minimal amount of (∼1 mL) of Et_2_O. The solid was transferred to a 20 mL glass scintillation vial and dried under vacuum overnight to afford 0.481 g of **3** to be used without further purification. Yield: 63%. The 1,4-dioxane content was quantified through a destructive NMR experiment in which dissolution of **3** in D_2_O affords liberation of the 1,4-dioxane. The amount of 1,4-dioxane was quantified via the inclusion of an acetone internal standard (Figure S10): (400 MHz, D_2_O, 24 °C) δ = 3.71 (s, dioxane), 2.23 (s), 2.20 (s, acetone).

### Preparation of Co(acac)_2_(dppBz), **2**

In an N_2_-filled glovebox, 0.750 g (2.9 mmol) of Co(acac)_2_ was transferred to a 20 mL scintillation vial containing a magnetic stir bar and dissolved in 5 mL of DCM. 1.30 g (2.9 mmol) of dppBz dissolved in 5 mL of DCM was added to the Co(acac)_2_ solution while stirring. The resulting dark red solution was stirred for 30 min and subsequently dried under vacuum. The solid was used without further purification to afford 1.95 g of **2**. Yield: 96% yield. ^1^H NMR (400 MHz, CD_3_CN, 24 °C) δ = 10.84 (br), 9.62 (br), 8.2–7.0 (m, br), 5.47 (s, DCM), 1.99 (s), 1.17 ppm (br).

### Protocol for Mass Spectrometry Sample Preparation

*Method A*. The following procedure was adapted from Hood et al.^32^ In an N_2_-filled glovebox, 5 mg of the analyte was massed into a vial and capped prior to removal from the glovebox. The sample was brought to the ESI-TOF mass spectrometer, where it was prepared for analysis. The vial was uncapped within a fume hood under ambient conditions, and sufficient LCMS-grade CH_3_CN was added to afford a concentration of 50 μg/mL. The solution was then transferred to a vial suitable for analysis.

*Method B*. Within an N_2_-filled glovebox, 10 mg of the analyte was transferred to a vial. To this vial was added a sufficient amount of LCMS grade CH_3_CN to afford a 10 mg/mL stock solution. Using the LCMS-grade CH_3_CN, the stock solution was diluted to 50 μg/mL. Of the diluted solutions, 100 μL aliquots were transferred to individual vials outfitted with a glass liner suitable for ESI-MS analysis and sealed with a septum cap. The septum cap was rigorously wrapped with Para film and placed inside a 20 mL scintillation vial prior to removal from the glovebox. The sample was brought to the mass spectrometer and removed from the outer scintillation vial immediately prior to analysis.

### Mass Spectrometry Analysis

Mass spectrometric measurements were conducted on an Agilent 6230 electrospray time-of-flight mass spectrometer (Agilent, Santa Clara, CA, USA) coupled to an Agilent 1260 Infinity II quaternary liquid chromatograph. Samples were run with a capillary voltage of 4000 V. Nitrogen was used as drying gas delivered at 10 L/min at a temperature of 325 °C, and the fragmenter voltage was set to 150 V. The mass range used was 100–3000 *m*/*z*. 0.5 μL of a 50 μg/mL sample was used for each injection, and samples were run in positive mode. Samples were injected in flow-through mode with a flow of 400 μL/min using an isocratic solvent composition with 100% acetonitrile. All other solvents used in the LC-System (e.g., needle wash solution) were acid and water free. LC-MS data were exported to mzData file format with MassHunter Workstation module Qualitative Analysis Navigator (Ver. B.08.00, Build 8.0.8208.0). Extracted ion chromatograms (EICs) were created using the exact mass of the compound of interest and a tolerance window of 10 ppm. Area under the curve (AUC) for each experiment was obtained by integrating each EIC with the Agile 2 integrating algorithm. Prior to data acquisition, the instrument was calibrated with a sodium formate solution, which resulted in an average error of 0.3 ppm over the 50–1200 *m*/*z* mass range recorded and a root-mean-square error of <1%. Isotopic patterns are simulated to confirm molecular identity.

### Catalytic Assay Procedure

All catalytic assays were performed using the following procedure, unless stated otherwise. The Parr reactor was initially purged with N_2_ for a minimum of 30 min. The pressure was then reduced under vacuum. The precatalyst solution, consisting of the precatalyst dissolved in tetraglyme, was injected into the main reservoir through the venting arm from a septum-sealed flask via cannula transfer. Similarly, 1-hexene was injected into the reservoir arm of the reactor via cannula transfer.

Once the precatalyst solution and 1-hexene are injected into their respective reservoirs, a flexible steel hose is attached to the closed reservoir arm and sample withdrawal arm. The steel hose was then purged with high-purity 1:1 H_2_:CO syngas through the side valve of the sample withdrawal arm. The reactor was pressurized to the desired pressure via the sample withdrawal arm with syngas, which was monitored by an electronic pressure transducer connected to a Parr 4871 process controller. Then the reactor was heated to the desired temperature while monitoring the pressure to ensure the pressure did not increase significantly over the target pressure. Once at the desired temperature, the valve connecting the main reservoir to the sample withdrawal arm was closed to reseal the reactor. The pressure was then reduced to ∼7 bar less than the desired pressure via the venting arm. The reaction was started by opening the top valve of the reservoir arm, followed by the lower valve to push the 1-hexene into the main reservoir and repressurizing the reactor to the desired pressure. Both valves were left open for the remainder of the experiment. The pressure was monitored and adjusted so that the pressure remained within 1 bar of the desired pressure for the duration of the experiment.

Once the assay was completed, the gas pressure regulator and upper valve on the sample withdrawal arm were closed. The sample withdrawal arm’s lower valve was opened to allow some of the catalyst solution to fill the small space in the arm. The lower valve was then closed before the side valve was opened to push out the catalyst solution into a 20 mL scintillation vial. The sample was then diluted in a 2:1 ratio with acetone in a GC/MS vial. The diluted sample was analyzed by GC-MS.

## Supporting Information Available

Corresponding NMR, EPR, and UV–vis spectra, mass spectrometry data, and GC-MS calibration methods (PDF)
