# Supplementary material for: Revisiting the Preparation and Catalytic Performance of a Phosphine-Modified Co(II) Hydroformylation Precatalyst
Source: J Am Chem Soc. 2024 Jul 2;146(28):19183–92. doi: 10.1021/jacs.4c04239 (PMC11258681; doi:10.1021/jacs.4c04239)
Supplement: Supplementary file 1 — ja4c04239_si_001.pdf [file ja4c04239_si_001.pdf]

Supplemental Materials for

Revisiting the Preparation and Catalytic Performance of a Phosphine-Modified Co(II) Hydroformylation Precatalyst

David R. Holzknecht,[a] Alexandra K. Van Alstine,[a] Brandon P. Russell,[b] David J. Vinyard,[b] Fabrizio Donnarumma[a] and Matthew B. Chambers[a]*

[a]Department of Chemistry, Louisiana State University, Baton Rouge, LA 70803-1804, United States.
[b]Department of Biological Sciences, Louisiana State University, Baton Rouge, LA 70803-1804, United States.

Table of Contents

^{1}H NMR spectra and ESI-TOF MS of Complex 1……………………………………………2

^{1}H NMR spectra and ESI-TOF MS of Complex 2……………………………………………8

^{1}H NMR spectra and ESI-TOF MS of Complex 3…………………………………………10

Determination of various contaminants influence on the characterization of 1………..…...13

Quantification of sample purity by ESI-TOF MS…………………………………….…….22

GC-MS calibration of hydroformylation products…………………………………….…..24

Hydroformylation Studies…………………………..…………………………………….25

Thermolysis Studies of 1………………………………………………………………..26

EPR Spectra of 1 and 2..……………………………………………………….…….28

^{1}H NMR spectra and ESI-TOF MS of Complex 1

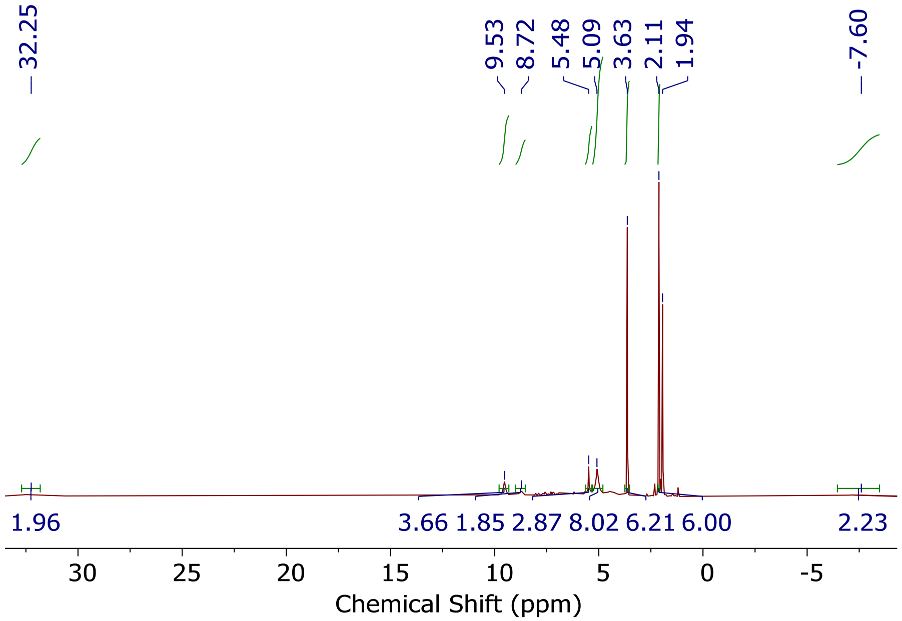

Figure S1A. ^{1}H NMR (400 MHz) spectrum of **1** prepared via method A in CD_3CN.

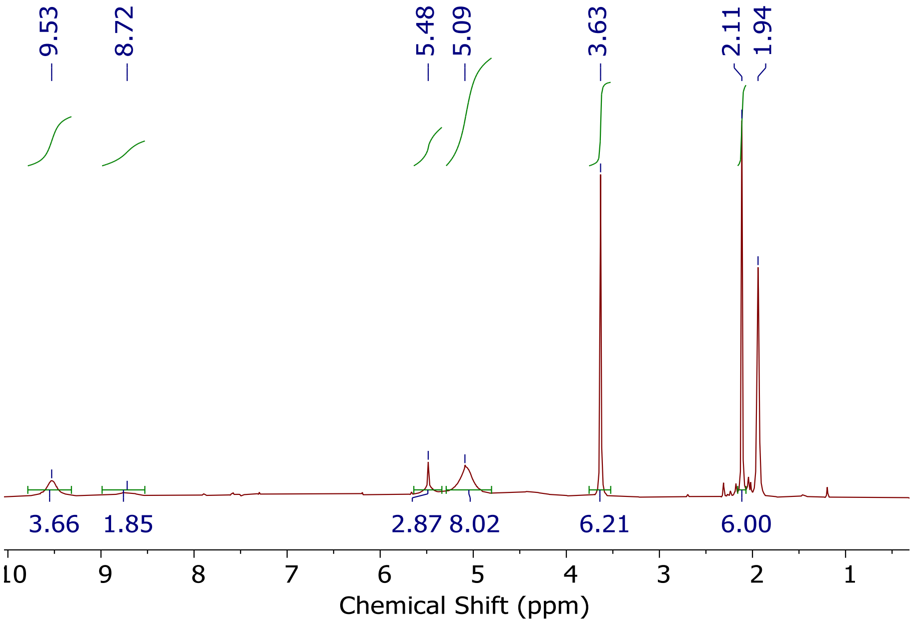

Figure S1B. ^{1}H NMR (400 MHz) spectrum of **1** prepared via method A in CD_3CN highlighting the 10-0 ppm range from Figure S1A. The resonance at δ 2.11 is assigned to the CH_3 groups of the acac ligand.

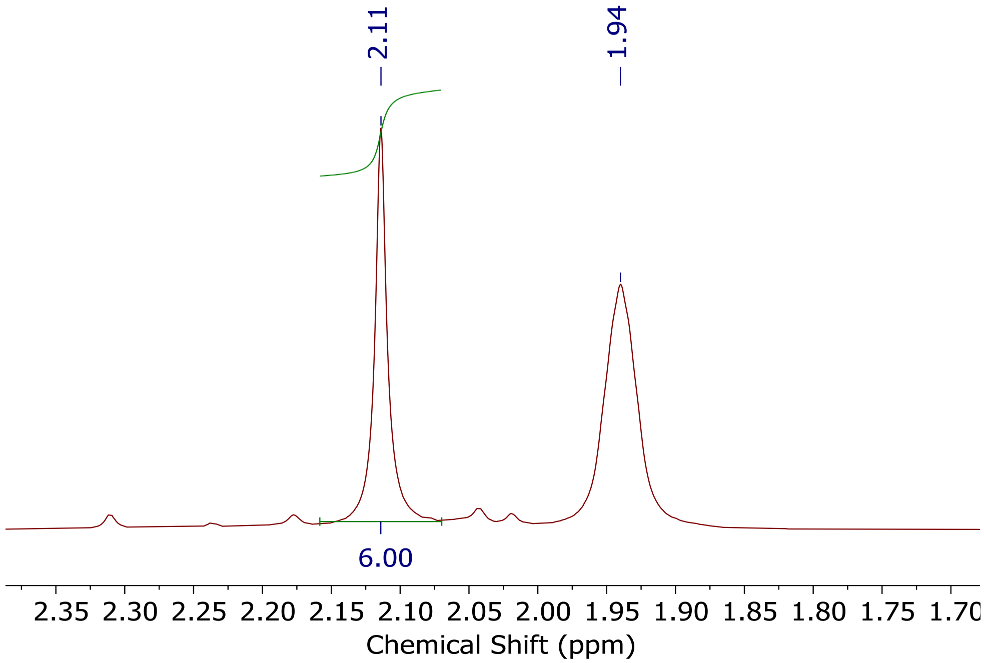

Figure S1C. ^{1}H NMR (400 MHz) spectrum of **1** prepared via method A in CD_3CN, specifically emphasizing the integration of the CH_3 groups of the acac ligand. Integrations of this resonance relative to the other resonances observed in the spectra are presented in Figures S1A and S1B. The resonance at δ 1.94 is the residual CD_2HCN.

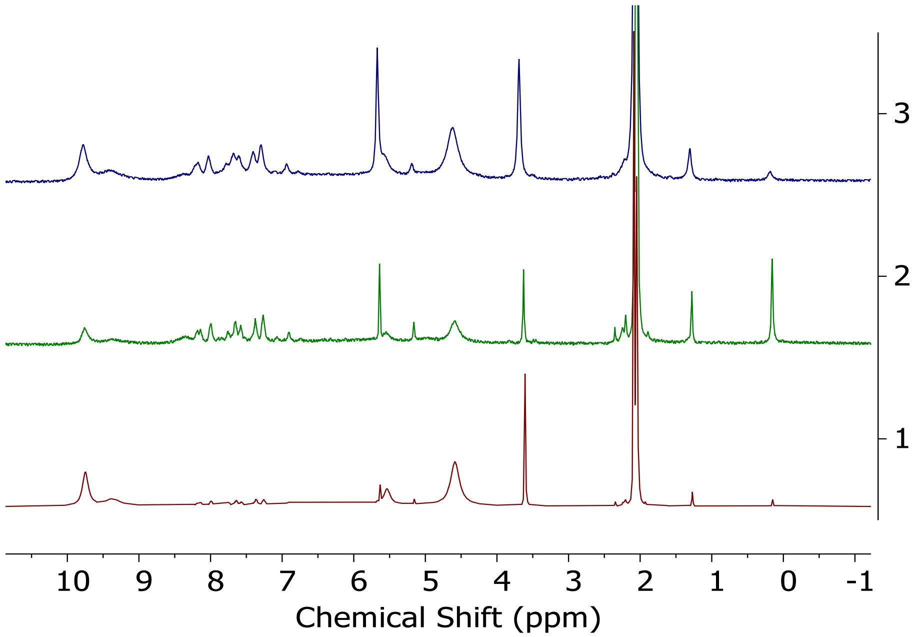

Figure S2. ^{1}H NMR (400 MHz) spectra of different batches of **1** prepared via method A in $(CD_3)_2CO$. As precatalysts, (Blue) produced 42.5% aldehyde in one hour. (Green) produced 52.5% aldehyde in one hour. (Red) produced 33.9% aldehyde in one hour.

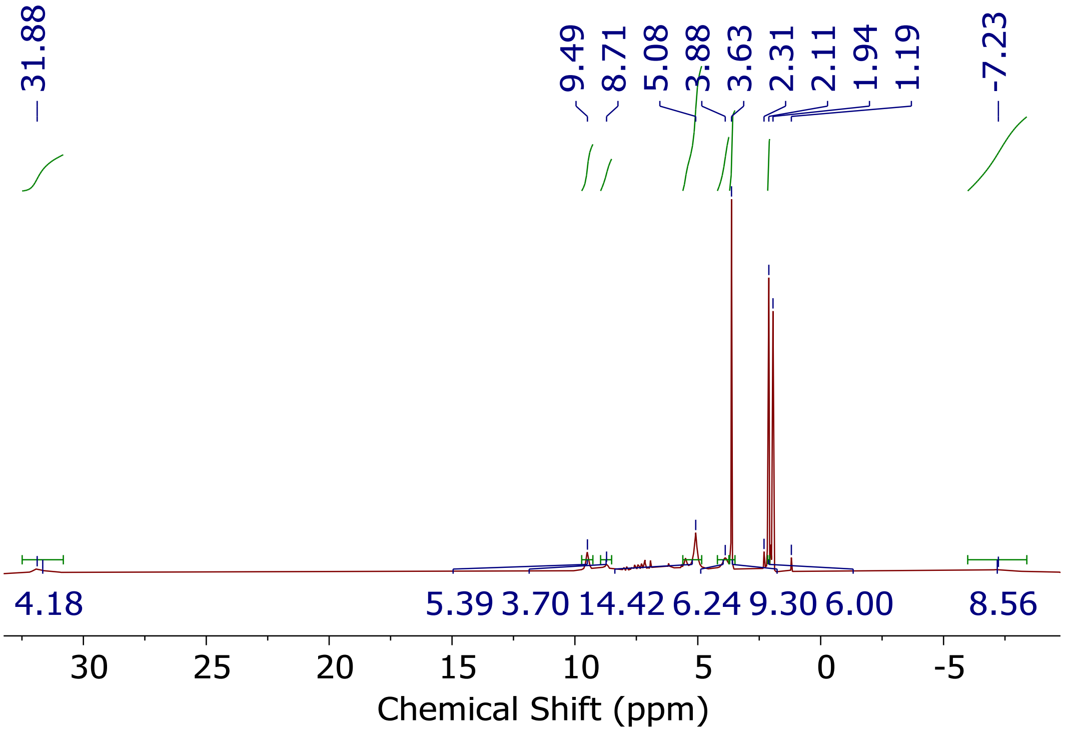

Figure S3A. ^{1}H NMR (400 MHz) spectrum of **1** prepared via method B in CD_3CN.

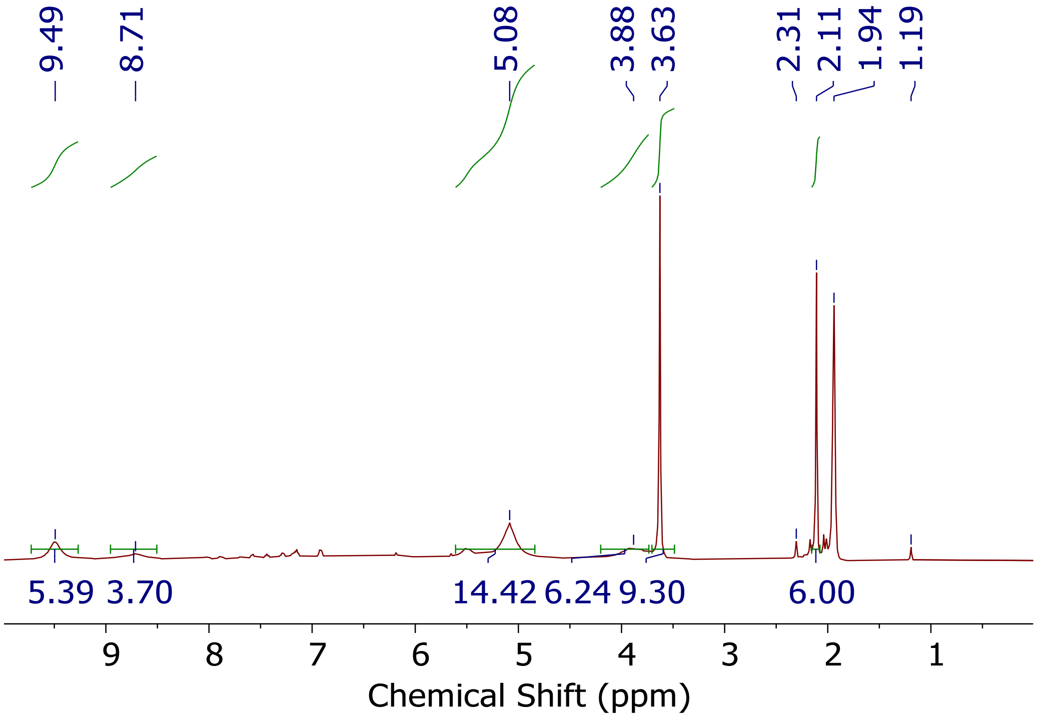

Figure S3B. ^{1}H NMR (400 MHz) spectrum of **1** prepared via method B in CD_3CN highlighting the 10-0 ppm range from Figure S1A. The resonance at δ 2.11 is assigned to the CH_3 groups of the acac ligand.

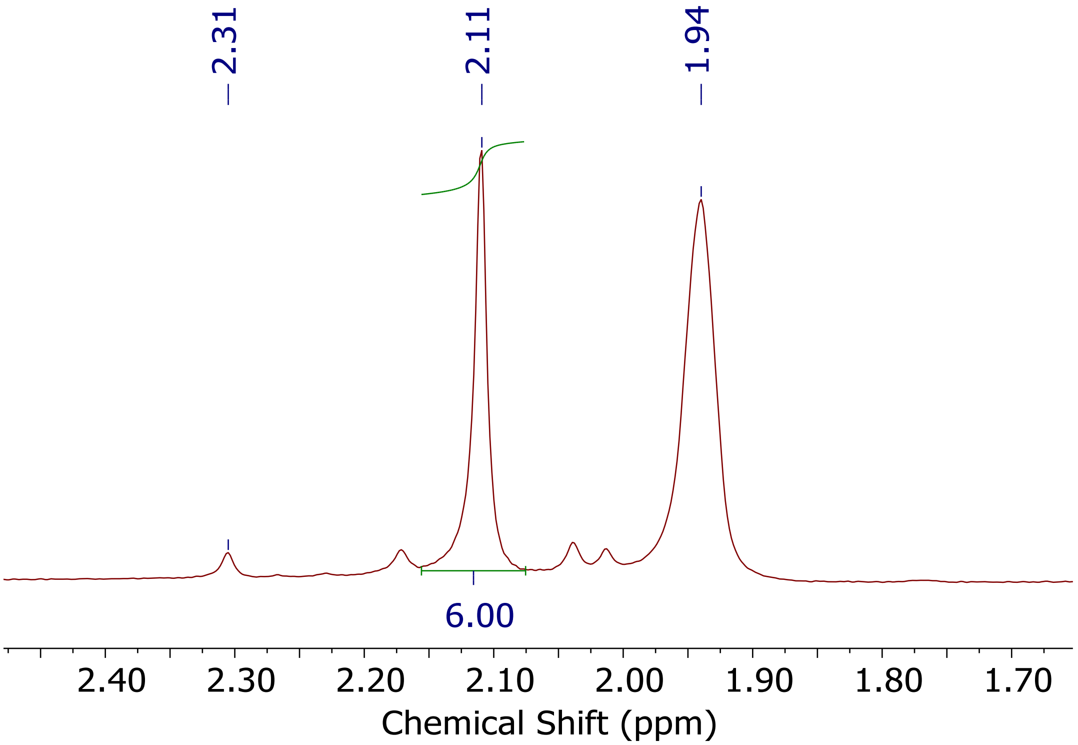

Figure S3C. ^{1}H NMR (400 MHz) spectrum of **1** prepared via method B in CD_3CN, specifically emphasizing the integration of the CH_3 groups of the acac ligand.

*In comparing the ^{1}H NMR spectra from Figure S1 and Figure S3, identification of the CH_3 resonance corresponding to impurity **2** is not tenable due to rapid exchange of the acac ligand. The relative integration of the acac resonance at 2.11 can be compared to the diagnostic resonance of **1** at δ 32 and –7.6 ppm, respectively. For samples with higher contamination of **2**, there is proportionally more acac in the sample than **1** and the ratio of the integration between the broad features of **1** and the resonance at δ 2.11 ppm diminishes. This is observed as samples prepared using the revised method B protocol (Figure S3) have a smaller ratio of acac to the broad features of **1**, indicating a higher purity level.

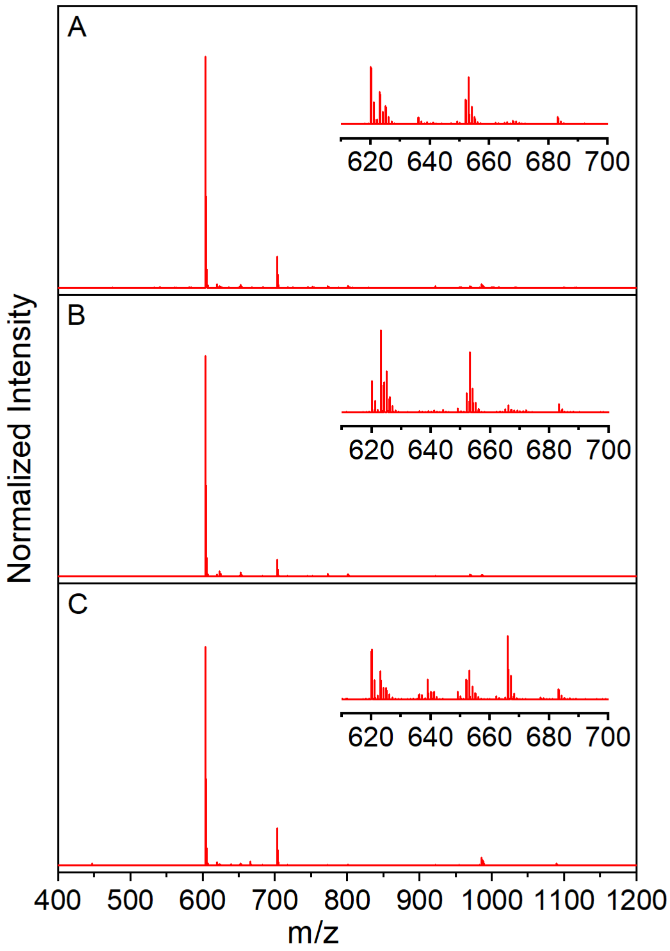

Figure S4. ESI-TOF mass spectra of three different batches of **1** prepared via method B in acetonitrile. The inset figures show the zoomed in 610-700 m/z region.

Table S1. Selected m/z values from Figure S4A for all m/z values ≥2% relative intensity.

Mass (m/z)	Relative Intensity (%)
604.1201	100
703.1602	14

Table S2. Selected m/z values from Figure S4B for all m/z values ≥2% relative intensity.

Mass (m/z)	Relative Intensity (%)
604.1145	100
703.1604	7

Table S3. Selected m/z values from Figure S4C for all m/z values ≥2% relative intensity.

Mass (m/z)	Relative Intensity (%)
604.1147	100
703.1606	17
986.1795	4

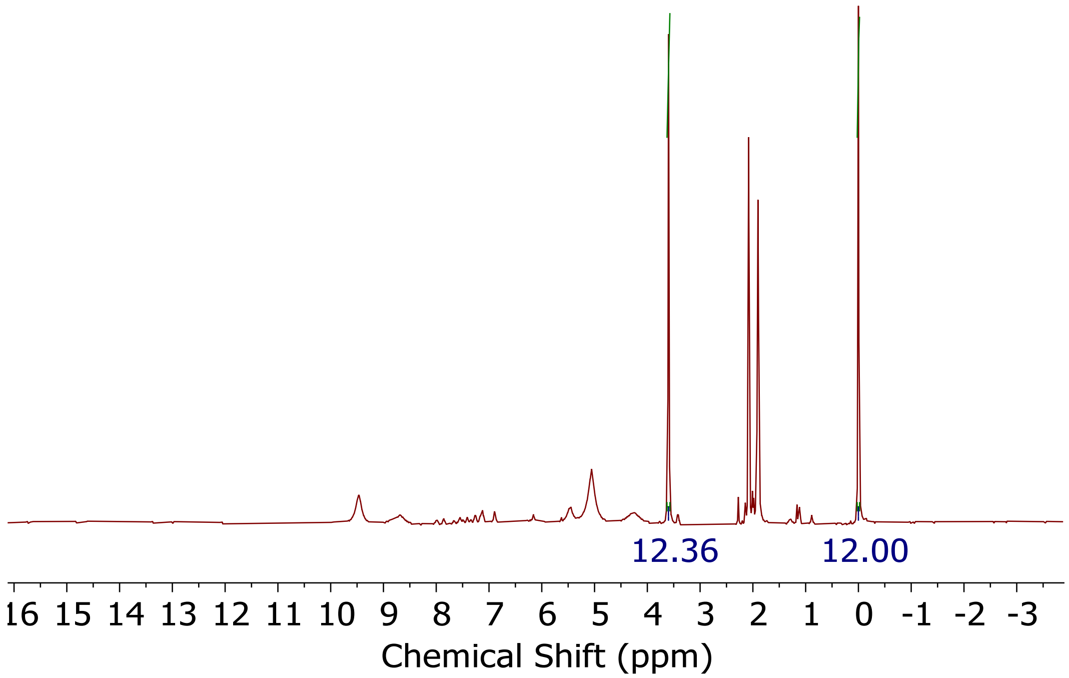

Figure S5. ^{1}H NMR (400 MHz) of **1** in CD_3CN with TMS as a standard with integrations below the TMS resonance and the dioxane resonance.

^{1}H NMR spectra and ESI-TOF MS of Complex 2

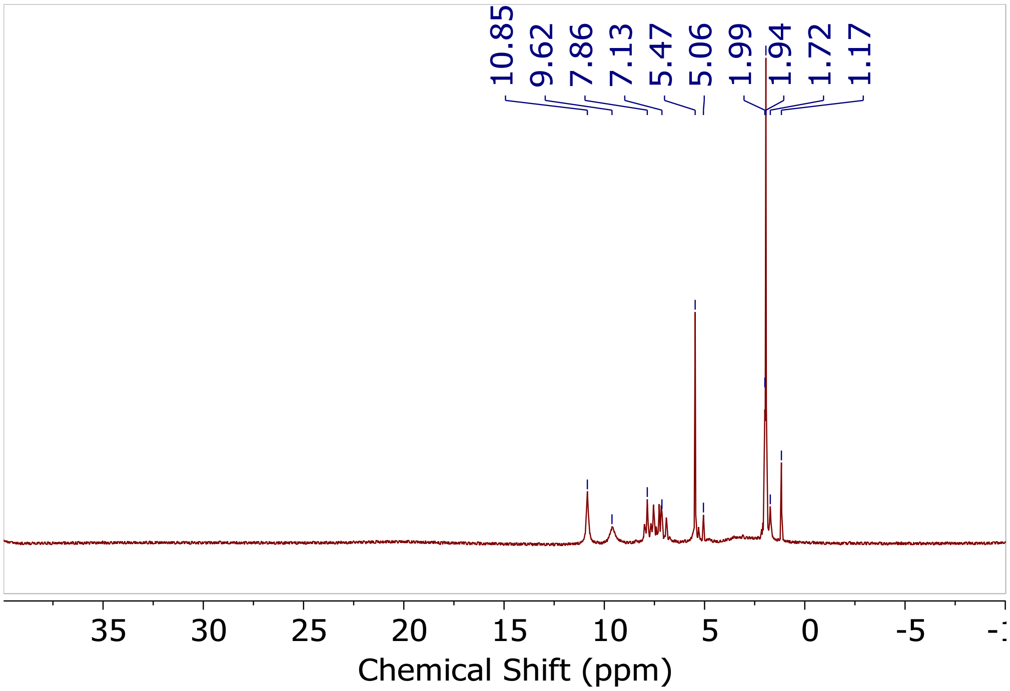

Figure S6A. ^{1}H NMR (400 MHz) spectrum of **2** in CD_3CN.

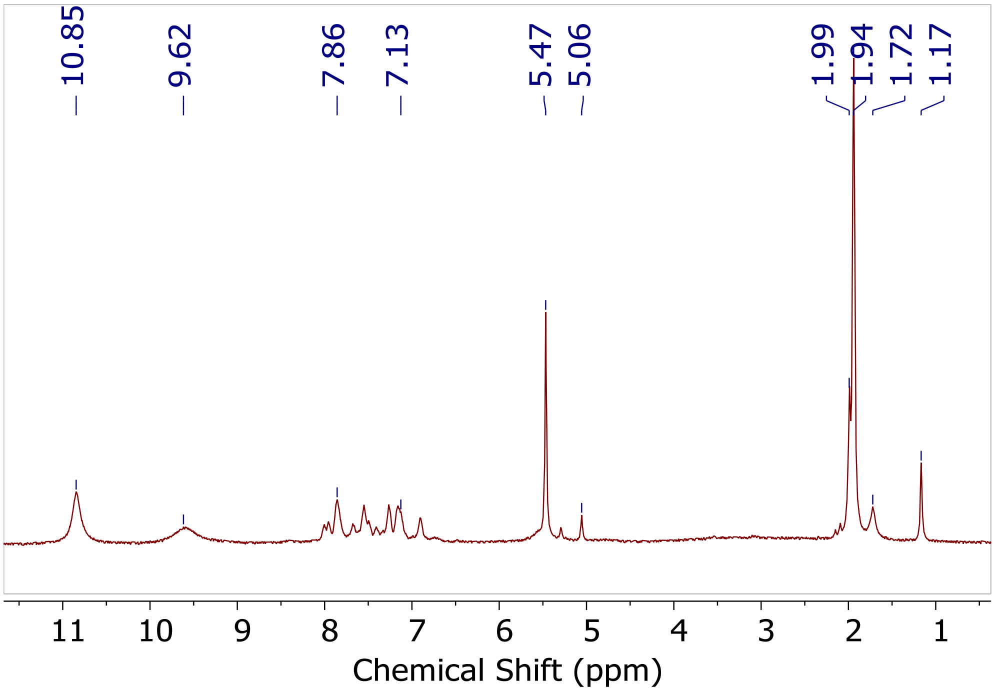

Figure S6B. ^{1}H NMR (400 MHz) spectrum of **2** in CD_3CN highlighting the 11.5-0.5 ppm range.

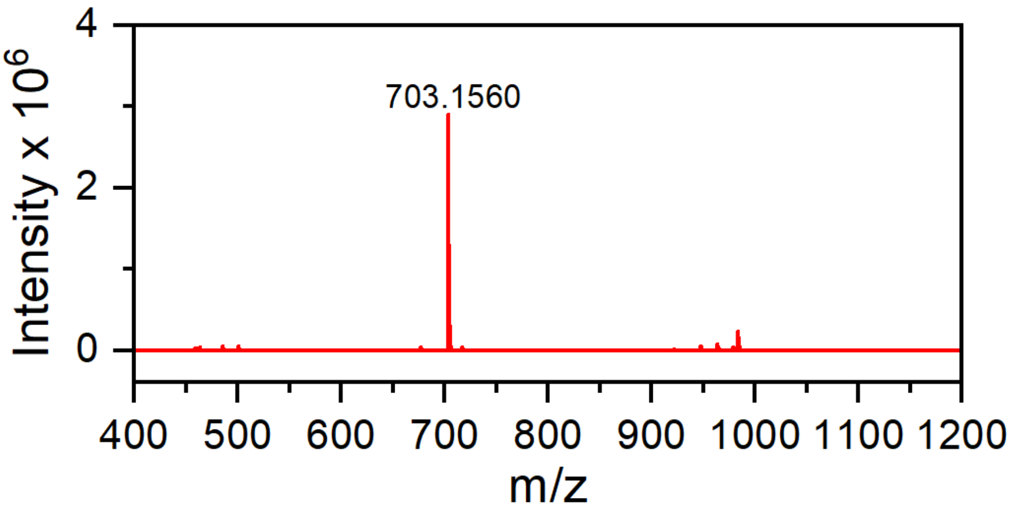

Figure S7. ESI-TOF mass spectrum of **2** in acetonitrile. $Co(acac)_2(dppBz)$ calc. mass = 703.1577 m/z

Table S4. Selected m/z values from Figure S7 for all m/z values ≥2% relative intensity.

Mass (m/z)	Relative Intensity (%)
703.1571	100
947.2517	2
963.2460	3
979.2486	2
983.1980	8
984.2046	5

^{1}H NMR spectra and ESI-TOF MS of Complex 3

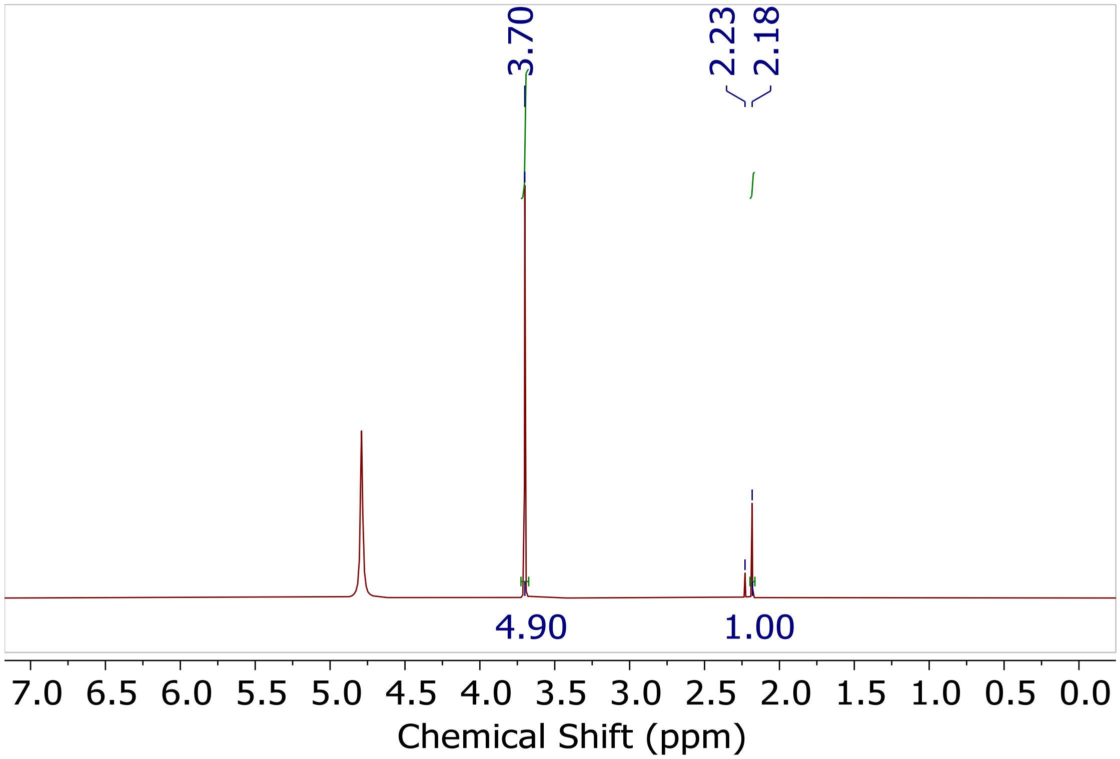

Figure S8. ^{1}H NMR (400 MHz) spectrum of **3** prepared via method A in D_2O with acetone as a standard. 3.9 dioxanes per cobalt were calculated from this spectrum.

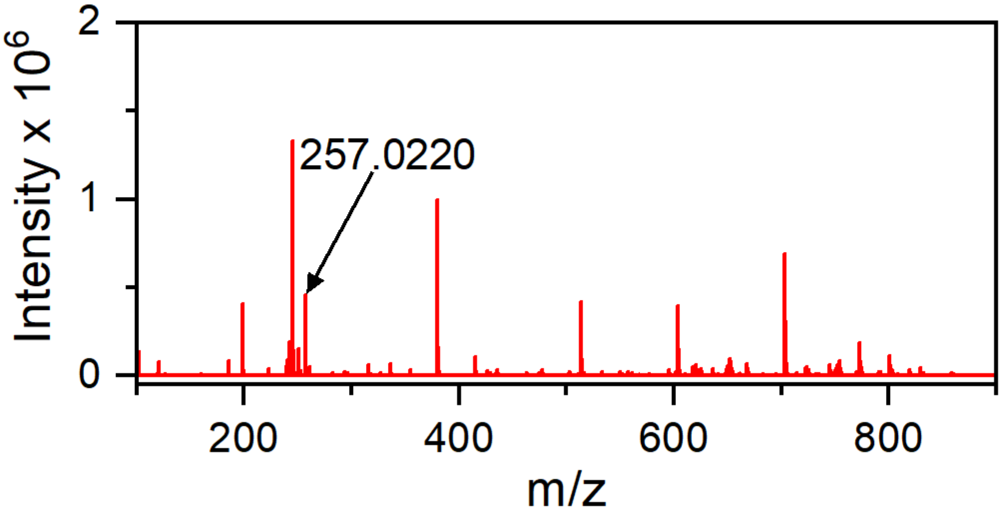

Figure S9. ESI-TOF mass spectrum of **3** prepared via method A in acetonitrile.

Table S5. Selected m/z values from Figure S9 for all m/z values ≥2% relative intensity.

**Mass (m/z)**	**Relative Intensity (%)**
245.1361	100
257.0220	34
380.1241	76
514.0551	31
604.1195	30
649.0555	3
652.0666	9
653.0913	7
668.0558	5
703.1658	54
723.0753	4
724.1082	5
751.1108	3
752.132	5
753.1382	3
754.0822	7
773.1007	14
801.1234	9

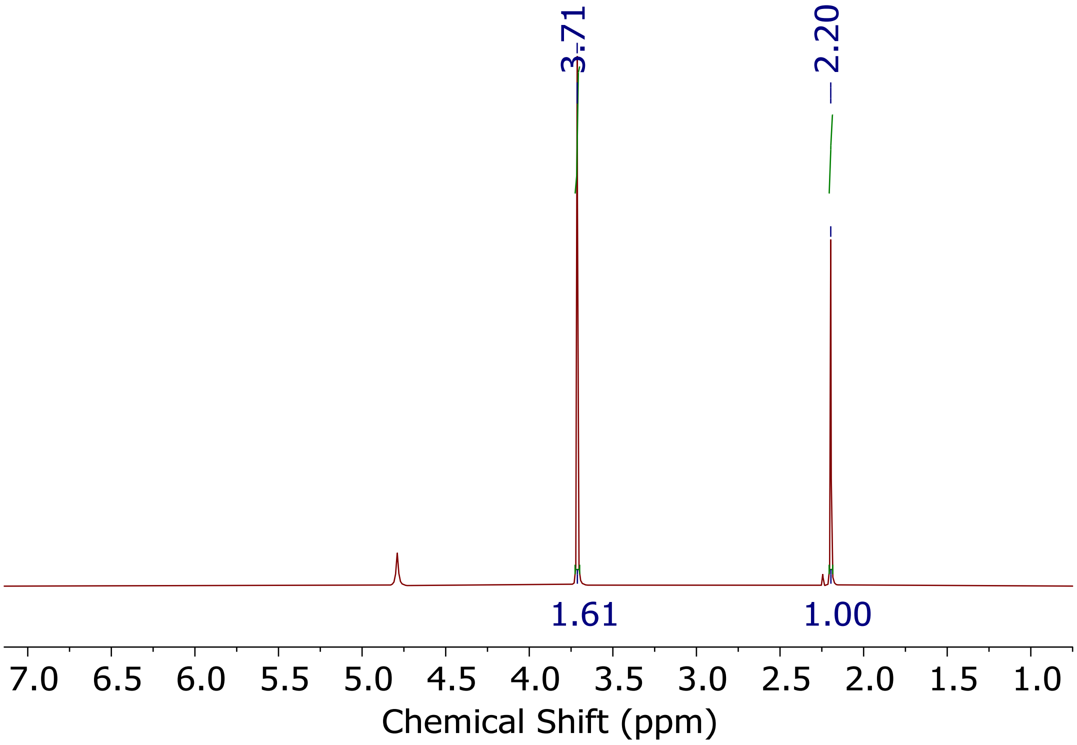

Figure S10. ^{1}H NMR (400 MHz) of **3** prepared via method B in D_2O with acetone as a standard. 4.5 dioxanes per cobalt were calculated from this spectrum.

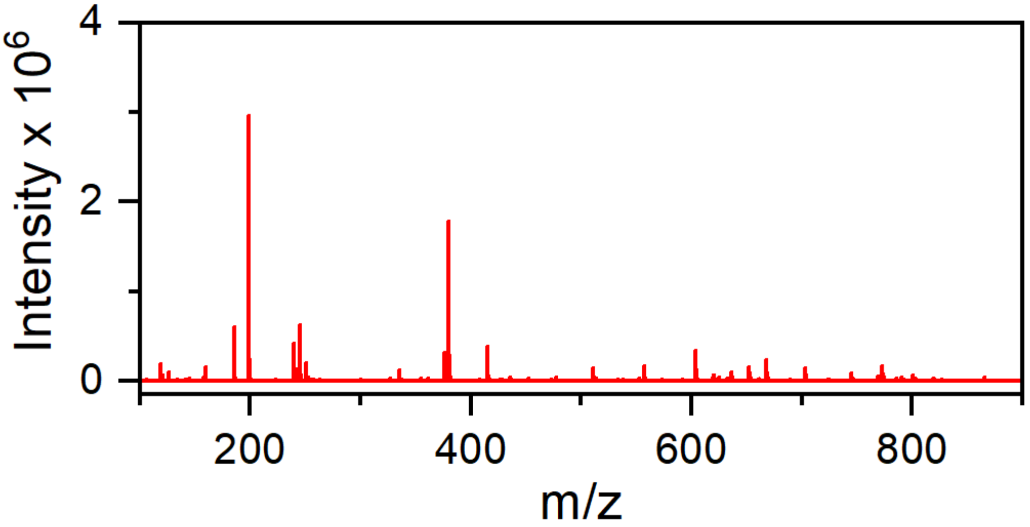

Figure S11. ESI-TOF mass spectrum of **3** prepared via method B in acetonitrile.

Table S6. Selected m/z values from Figure S11 for all m/z values ≥2% relative intensity.

Mass (m/z)	Relative Intensity (%)
199.0049	100
240.0304	14
245.1359	20
251.1572	7
375.9899	10
380.1238	60
415.0011	13
604.1193	12
636.0974	4
652.0811	5
653.0912	3
668.0556	8
703.1657	5
745.0604	3
773.1006	6
1032.206	4

Determination of various contaminants influence on the characterization of 1

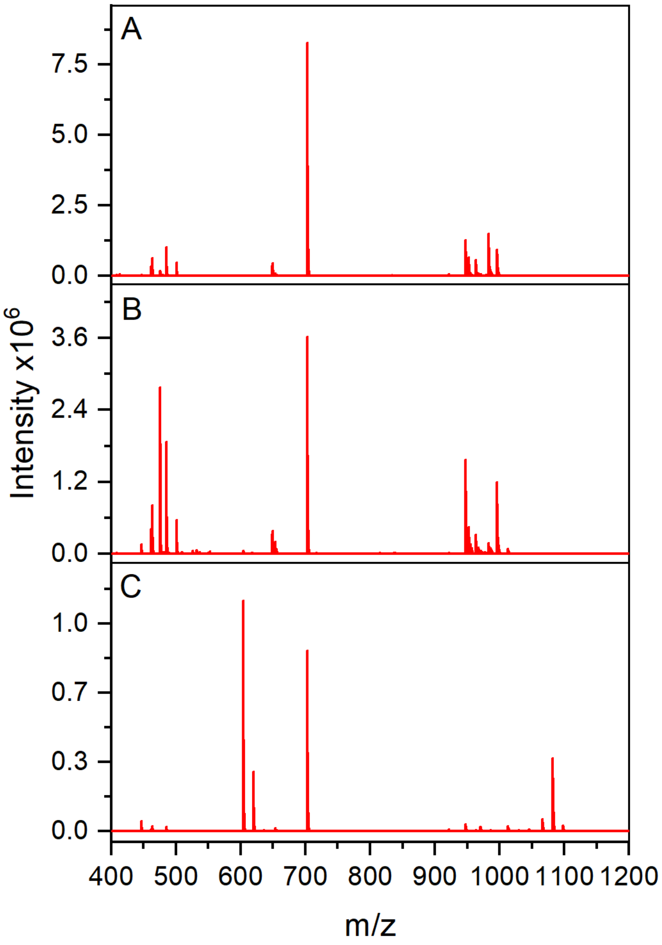

Figure S12. ESI-TOF mass spectra of **1** in different solvent conditions. (A) 80/20 MeOH and 0.1% formic acid in water. (B) 60/40 acetonitrile and 0.1% formic acid in water. (C) acetonitrile.

Table S7. Selected m/z values from Figure S12A for all m/z values ≥2% relative intensity.

Mass (m/z)	Relative Intensity (%)
**461.1656**	4
463.1491	7
485.1231	12
501.1195	5
649.1192	5
703.1575	100
704.1608	47
947.2522	15
948.2578	10
951.2072	2
952.215	8

963.2464	7
964.2605	4
983.1984	18
984.205	12
986.1837	2
987.1918	2
996.2154	12
997.2105	9

Table S8. Selected m/z values from Figure S12B for all m/z values ≥2% relative intensity.

**Mass (m/z)**	**Relative Intensity (%)**
461.1666	11
463.1502	23
475.6033	74
485.1242	50
501.1207	16
649.1207	10
653.0982	6
703.1591	100
947.2542	44
948.2599	29
951.2093	3
952.2171	12
955.2081	5
963.2485	9
983.2006	5
986.1858	3
996.2176	34
997.2127	23

Table S9. Selected m/z values from Figure S12C for all m/z values ≥2% relative intensity.

**Mass (m/z)**	**Relative Intensity (%)**
447.1454	4
604.1236	100
620.1170	25
703.1726	78
947.2518	3
970.2132	2
1013.1994	2
1066.2468	5
1082.2520	32
1098.2416	3

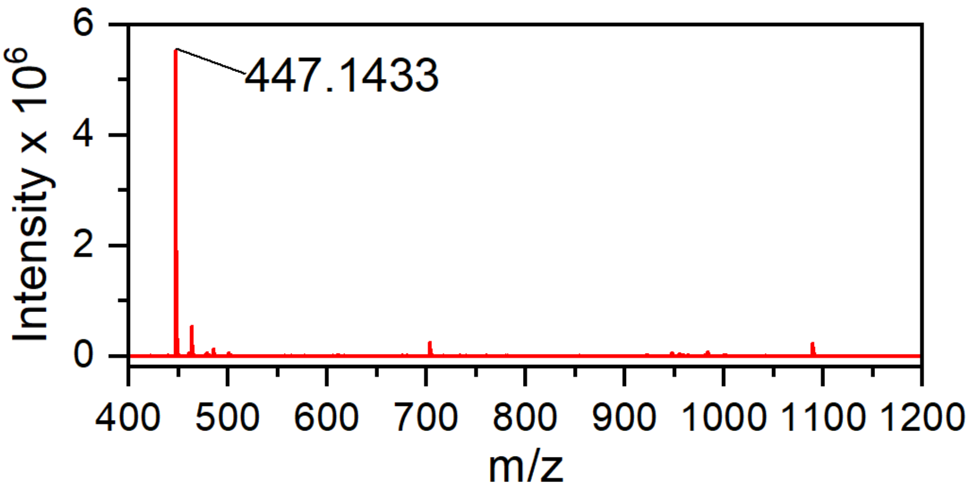

Figure S13. ESI-TOF mass spectrum of the precipitate that forms upon treatment of **1** with an acetonitrile/formic acid mixture. The m/z value of 447.1433 corresponds to $[dppBzH]^+$ (calc. = 447.1431 m/z).

Table S10. Selected m/z values from Figure S13 for all m/z values ≥2% relative intensity.

**Mass (m/z)**	**Relative Intensity (%)**
338.3420	4
447.1433	100
448.1459	34
449.1497	5
463.1383	10
703.1585	5
1089.237	4

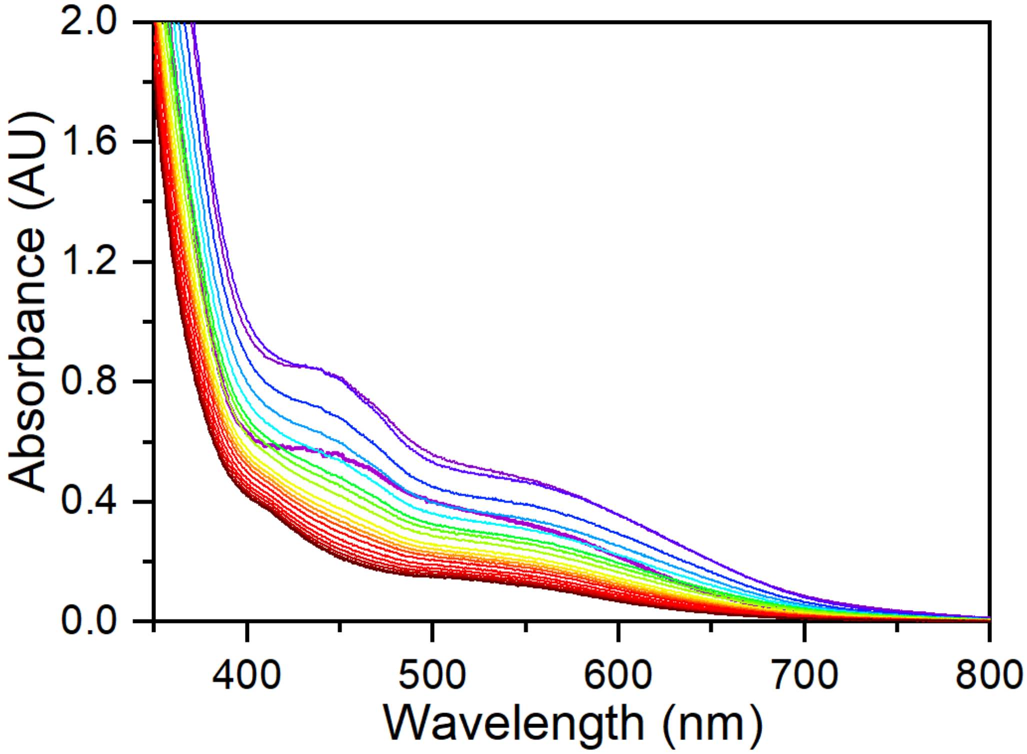

Figure S14. UV-vis spectra of 0.5 mM **1** in ACN in air. Time between scans = 5 minutes for the first hour and 10 minutes for the second hour.

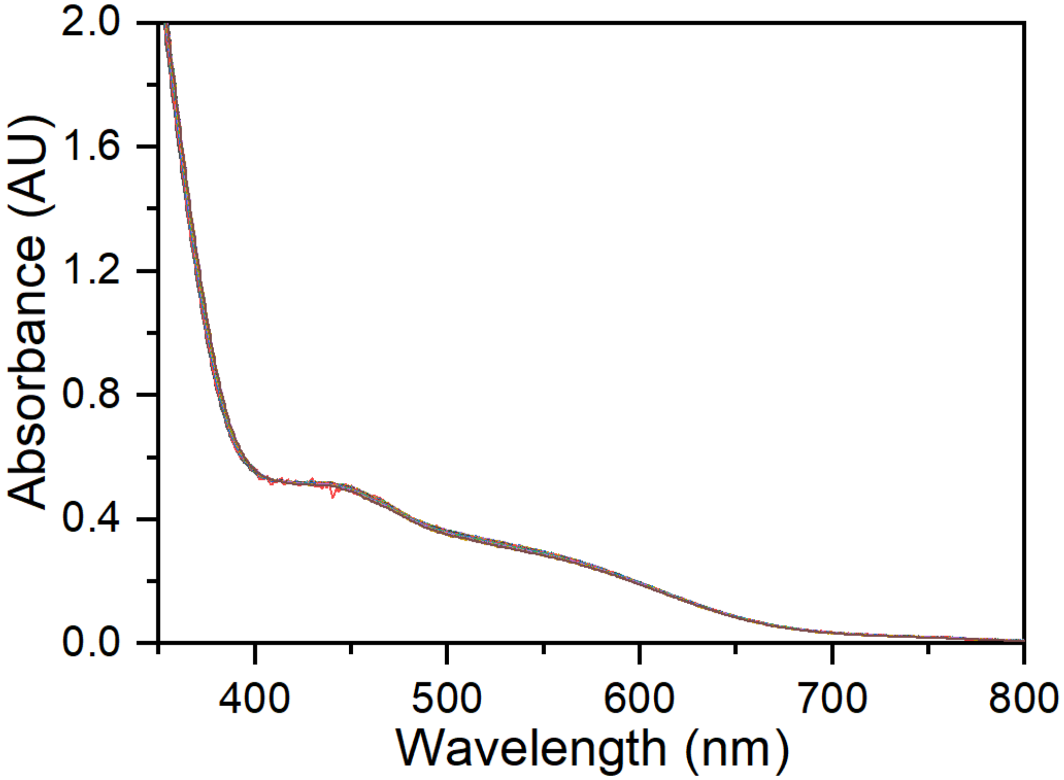

Figure S15. UV-vis spectra of 0.5 mM **1** in ACN under N_2**.** Time between scans = 5 minutes for the first hour and 10 minutes for the second hour.

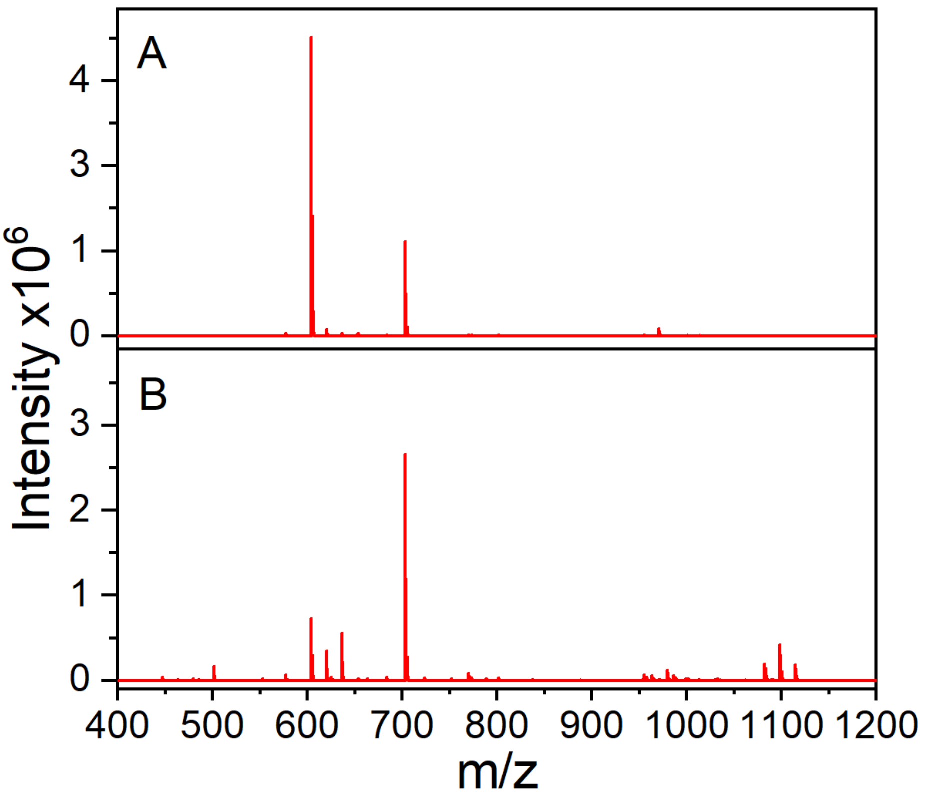

Figure S16. ESI-TOF mass spectra in acetonitrile of (A) **1** (B) The same sample of **1** but 30 minutes after the septum was pierced for the first sample.

Table S11. Selected m/z values from Figure S16A for all m/z values ≥2% relative intensity.

Mass (m/z)	Relative Intensity (%)
604.1258	100
703.1600	31

Table S12. Selected m/z values from Figure S16B for all m/z values ≥2% relative intensity.

Mass (m/z)	Relative Intensity (%)
604.1245	28
620.1180	13
636.1035	21
703.1585	100
979.2501	5
1082.2530	7
1098.2430	16
1114.2350	7

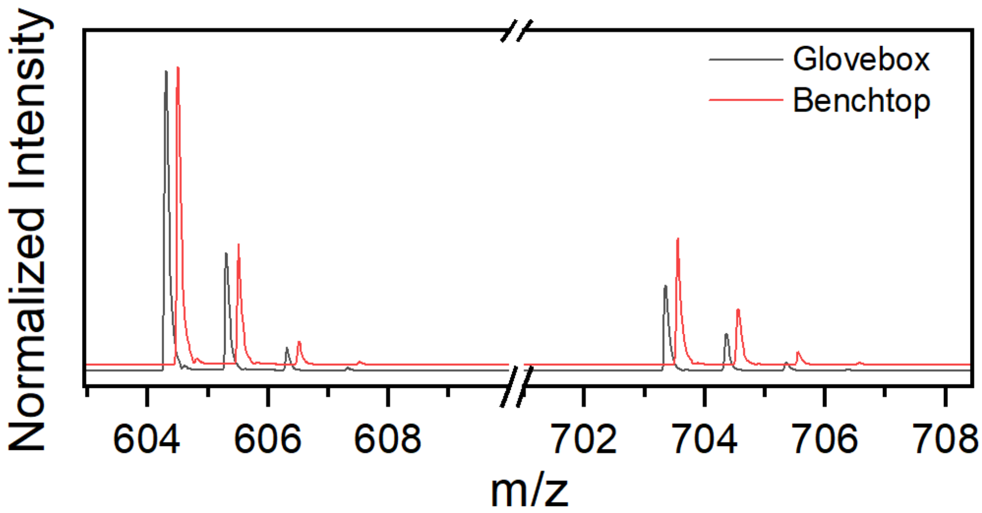

Figure S17. ESI-TOF mass spectra of **1** in acetonitrile where the sample was prepared (red trace) in a fume hood (black trace) in an N_2-filled glovebox. An xy offset was applied for visual clarity.

Table S13. Selected m/z values from Figure S17 (Benchtop) for all m/z values ≥2% relative intensity.

Mass (m/z)	Relative Intensity (%)
604.1207	100
620.1140	18
636.1139	3
703.1692	43
970.2084	2
1082.2470	4

Table S14. Selected m/z values from Figure S17 (Glovebox) for all m/z values ≥2% relative intensity.

Mass (m/z)	Relative Intensity (%)
604.1208	100
703.1694	29
970.2090	7

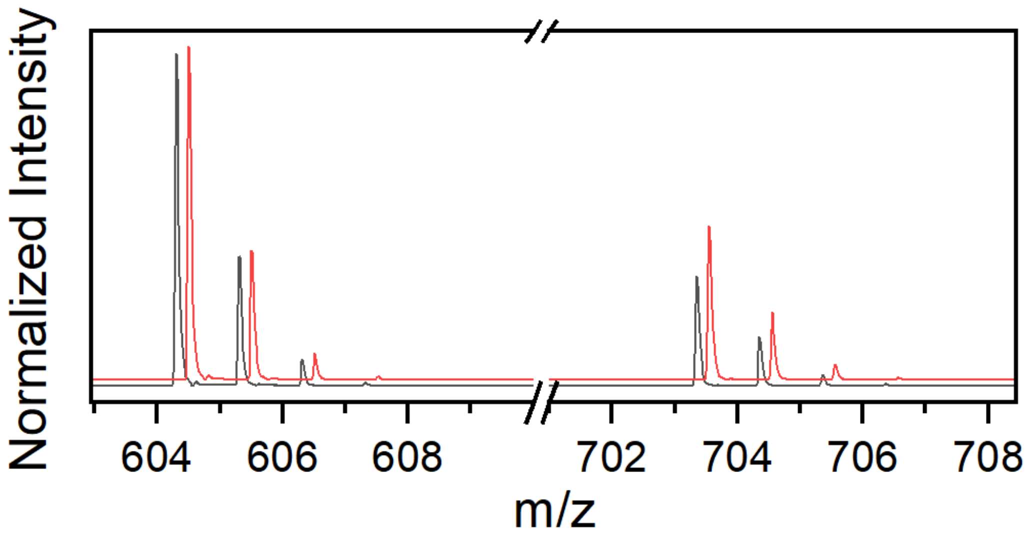

Figure S18. ESI-TOF mass spectra of **1** prepared from (red trace) **3** prepared via method A (black trace) **3** prepared via method B. An xy offset was applied for visual clarity.

Table S15. Selected m/z values from Figure S18 (method A) for all m/z values ≥2% relative intensity.

Mass (m/z)	Relative Intensity (%)
604.1174	100
703.1634	46
970.2129	3

Table S16. Selected m/z values from Figure S18 (method B) for all m/z values ≥2% relative intensity.

Mass (m/z)	Relative Intensity (%)
604.1172	100
703.1631	43
970.2125	2

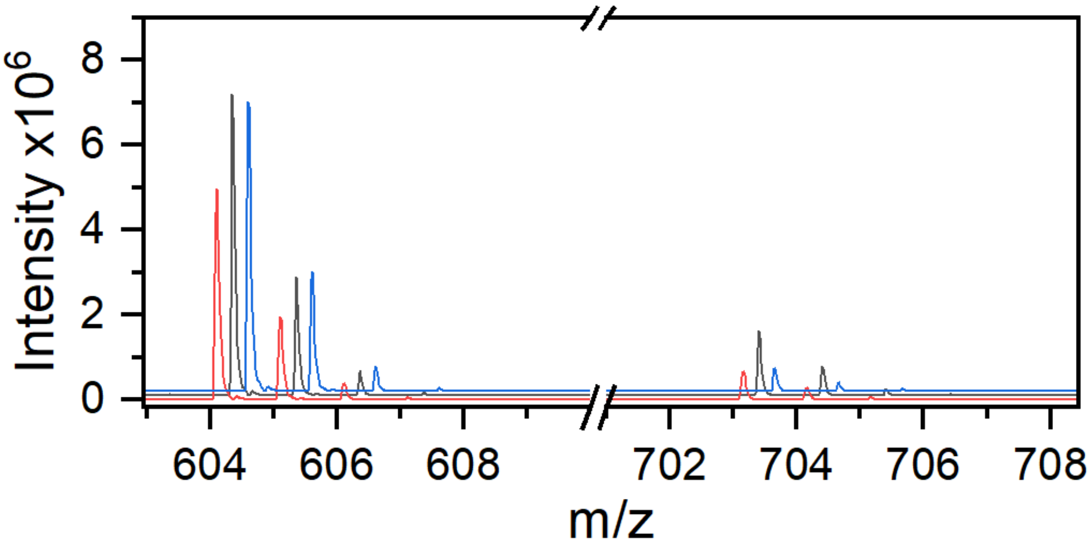

Figure S19 ESI-TOF MS of three different batches of **1** synthesized via method B. An xy offset was applied for visual clarity.

Table S17. Selected m/z values from Figure S19 (red trace) for all m/z values ≥2% relative intensity.

Mass (m/z)	Relative Intensity (%)
604.1173	100
703.1634	14

Table S18. Selected m/z values from Figure S19 (black trace) for all m/z values ≥2% relative intensity.

Mass (m/z)	Relative Intensity (%)
604.1173	100
703.1634	21
970.2130	2

Table S19. Selected m/z values from Figure S19 (blue trace) for all m/z values ≥2% relative intensity.

Mass (m/z)	Relative Intensity (%)
604.1173	100
703.1634	8

Quantification of sample purity by ESI-TOF MS

Table S20. Uncalibrated LC-MS analysis of **1** prepared via method A to determine its purity qualitatively

**Trial**	**$Area_{604}$ (counts)**	**$Area_{703}$ (counts)**	**Uncalibrated %1 ($Area_{604}$/($Area_{604}$+$Area_{703}$)*100)**
**1**	2.9 x 10^7	0.74 x 10^7	80
**2**	2.3 x 10^7	0.66 x 10^7	78
**3**	2.6 x 10^7	0.61 x 10^7	81
**Std. Dev.**	0.3 x 10^7	0.07 x 10^7	1

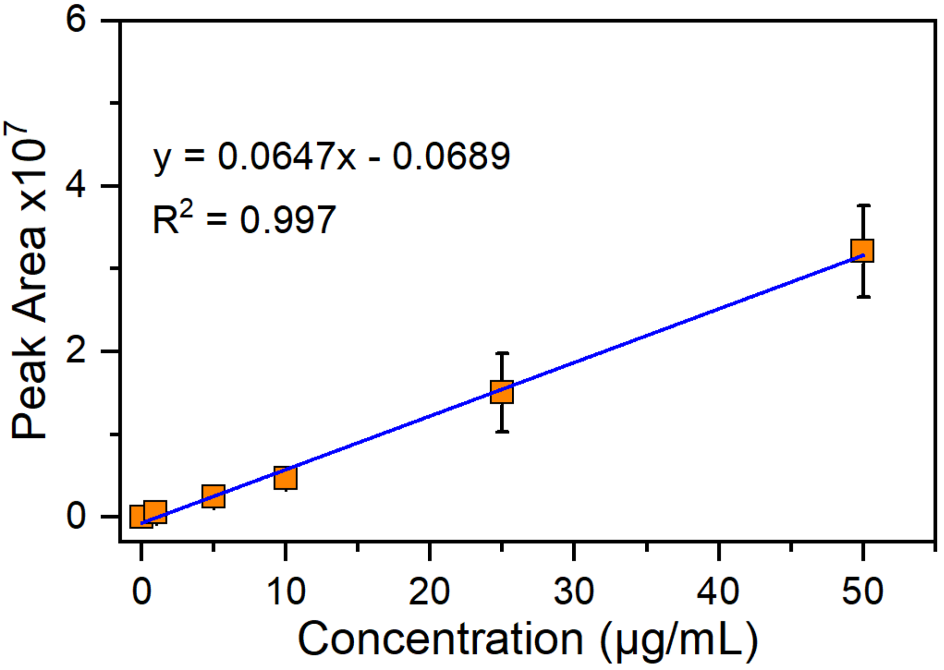

Figure S20. ESI-TOF MS calibration curve for **1** in acetonitrile using the peak area of 604.1120 m/z.

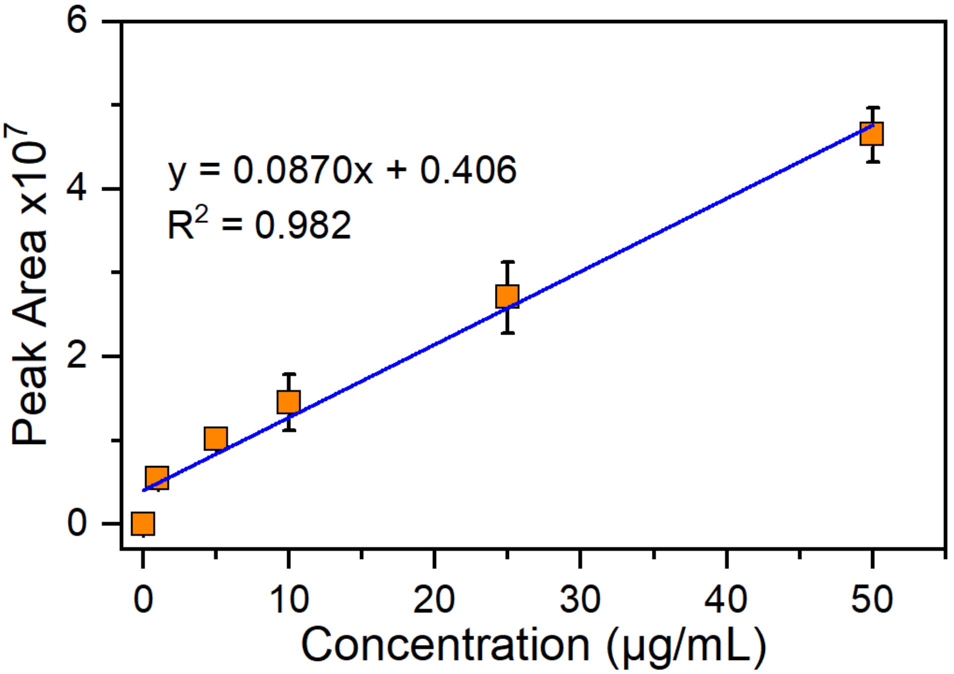

Figure S21. ESI-TOF MS calibration curve for **2** in acetonitrile using the peak area of 703.1560 m/z.

Table S21. Quantification of **1**'s purity by LC-MS analysis

Trial	$Area_{604}$ (counts)	$Area_{703}$ (counts)	Uncalibrated %1	[1] (µg/mL)	[2] (µg/mL)	Calibrated %1 ([1]/([1]+[2])*100)
**1**	$2.92x10^7$	$0.23x10^7$	94	46	<1	>99
**2**	$2.92x10^7$	$0.23x10^7$	94	46	<1	>99
**3**	$2.43x10^7$	$0.23x10^7$	93	38	<1	>99
**Std. Dev.**	$0.28x10^7$	$3.14x10^4$	1	5	<1	<1

GC-MS calibration of hydroformylation products

After a sample is taken from the Parr reactor, 0.5 mL of the sample is diluted with 1 mL of acetone before being taken to the GC-MS to be analyzed. To determine the concentration of each hydroformylation product, the response factor (Rf) of each analyte relative to heptane was determined and is shown in Table SC. The relative Rf values can be plugged into equation S1 to solve for the concentration of each product. Since hexane's retention time is less than our solvent cutoff (1.7 minutes) the concentration of hexane must be inferred from the rest of the data set. The concentration of hexane is determined by adding all of the concentrations of products together and subtracting it from 1 M. Since our sample should be 1 M, this means that hexane is designated as all the "missing" product.

$$Concentration\ of\ Analyte = \frac{0.033\ M * Area_{analyte} * 3}{Rf_{analyte} * Area_{heptane}} \qquad \text{Equation (S1)}$$

Table S22. 1-Hexene hydroformylation products and their GC-MS retention time and response factors relative to heptane

Product	Retention Time (min)	Rf
**1-Hexene**	2.040	0.61
**2-Hexene**	2.154/2.239	1.06
**2-Ethylpentanal**	7.695	0.95
**2-Methylhexanal**	7.992	0.95
**Heptanal**	10.039	1.15

Hydroformylation Studies

Table S23. Hydroformylation of 1-hexene using mixed pre-catalysts[a]

Mono:Bis	Yield (%)	Aldehydes (%)	*Iso*hexenes (%)	Hexane (%)
**>99:1**	68 ± 3	43 ± 3	15 ± 1	10 ± 2
**3:1**	63 ± 2	38 ± 5	16 ± 3	9 ± 8
**1:1**	47 ± 6	19 ± 8	9 ± 2	18 ± 6
**1:3**	58 ± 2	29 ± 7	12 ± 3	16 ± 9
**<1:99**	32 ± 5	5 ± 5	3 ± 1	24 ± 2

[a]1 mM [Co], 1 M (11.2 mL) 1-hexene, 0.1 M (1.3 mL) heptane standard, 77.5 mL tetraglyme, and 1:1 H_2:CO. Samples were taken 1 hour after addition of 1-hexene to the reaction mixture.

Table S24. Hydroformylation of 1-hexene with $Co_2(CO)_8$, **1**, and **3** as precatalysts.[a]

Precatalyst	Added Ligand	Yield (%)	Aldehydes (%)	*l:b*	*Iso*hexenes (%)	Hexane (%)
**[Co(acac)(dppBz)]BF_4**	N/A	68	43	1.1	15	10
**[Co(acac)(dioxane)$_4$]BF_4**	dppBz	73	53	1.1	19	5
**Co_2CO_8**	N/A	83	57	1.1	21	5
**Co_2CO_8**	dppBz	28	0	N/A	2	26

[a]1 mM [Co], 1 mM ligand, 1 M (11.2 mL) 1-hexene, 0.1 M (1.3 mL) heptane standard, 77.5 mL tetraglyme, and 1:1 H_2:CO. Samples were taken 1 hour after addition of 1-hexene to the reaction mixture.

Thermal Degradation Studies for Precatalyst 1

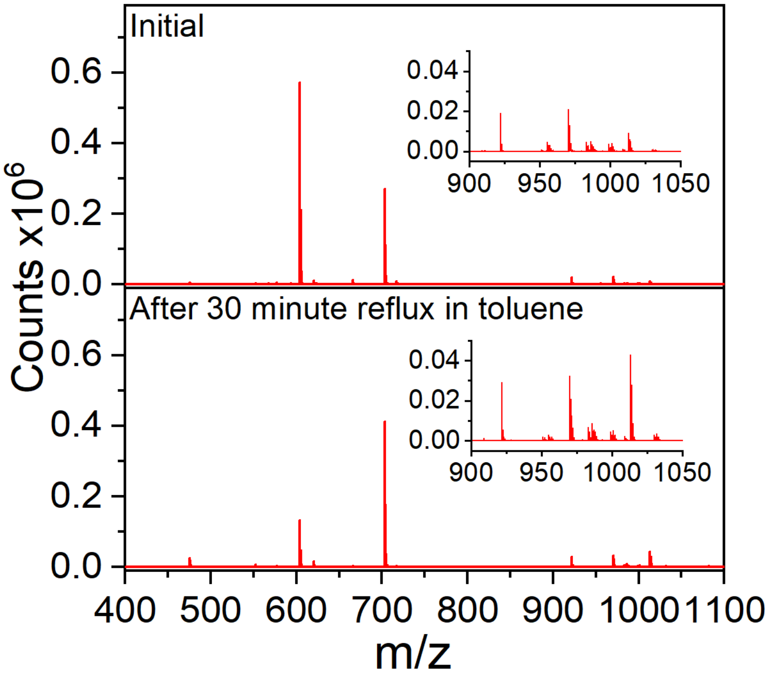

Figure S22. ESI-TOF MS data collected for a 1.3 mM solution of **1** in toluene refluxed under N_2 for 30 minutes. Top spectra for the sample pre-reflux; Bottom spectra for sample post-reflux. As quantified in Table S25, ~10% increase in the signal corresponding to impurity **2** is observed after reflux. Additionally, numerous new features between 900-1050 m/z are observed, indicating that the primary decomposition product of thermolysis of **1** is not complex **2** generated from ligand scrambling.

Table S25. Quantitative analysis of the ESI-TOF MS data (Figure S22) for the thermolysis of **1** in toluene.

Complex	Initial µmols in ESI-TOF MS sample	Final µmols in ESI-TOF MS sample	Change in µmols
**1**	0.0635	0.0145	0.049
**2**	0.0089	0.0143	0.0054

The data presented in Table S25 show that 0.049 µmol of **1** decomposed upon heating to generate an additional 0.0054 µmol of **2**. This is an empirical 11% yield of **2**. Considering that 2 equiv of **1** would be needed to generate 1 equiv of **2**, a maximum theoretical yield of **2** would be 50%.

Stoichiometry for the theoretical conversion of **1** to **2** upon heating:
2[Co(acac)(dppBz)]BF_4 → Co(acac)$_2$(dppBz) + *"[Co(dppBz)][BF$_4$]$_2$"*

The *"[Co(dppBz)][BF$_4$]$_2$"* species is neither observed nor proposed to be a stable complex, but rather represents the stoichiometric balance remaining after the production of **2**.

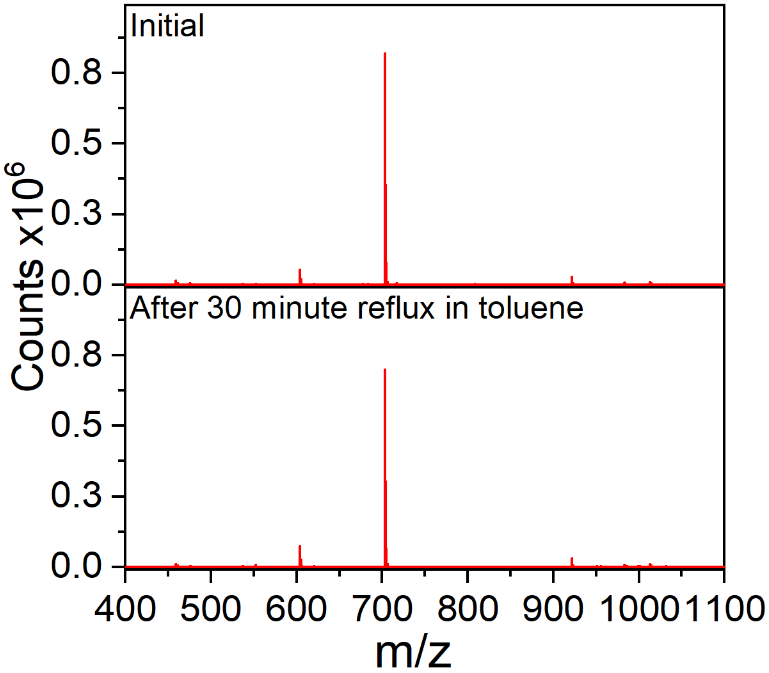

Figure S23. ESI-TOF MS data collected for a 1.3 mM solution of **2** in toluene refluxed under N_2 for 30 minutes. Top spectra for the sample pre-reflux; Bottom spectra for sample post-reflux. No degradation of **2** is observed under these conditions. No growth of signals between 900-1050 m/z is observed, as was seen upon heating **1** (Figure S22).

EPR Spectroscopy of 1 and 2

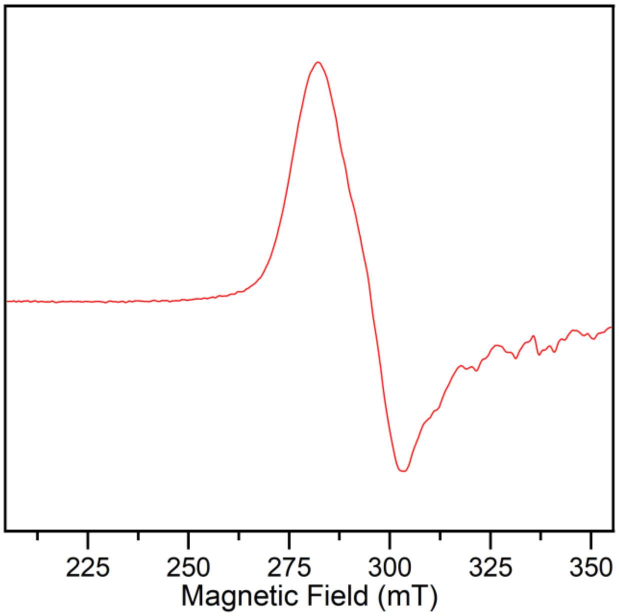

Figure S24. EPR spectrum of [Co(acac)(dppBz)]BF_4 in a frozen toluene solution. The EPR spectrum was collected at 77 K using 1 mW microwave power, operating at 9.43 GHz. The spectrum was broad and nearly isotropic with g_{iso} = 2.30. No hyperfine interactions from the ^{59}Co or ^{31}P nuclei were observed at this temperature.

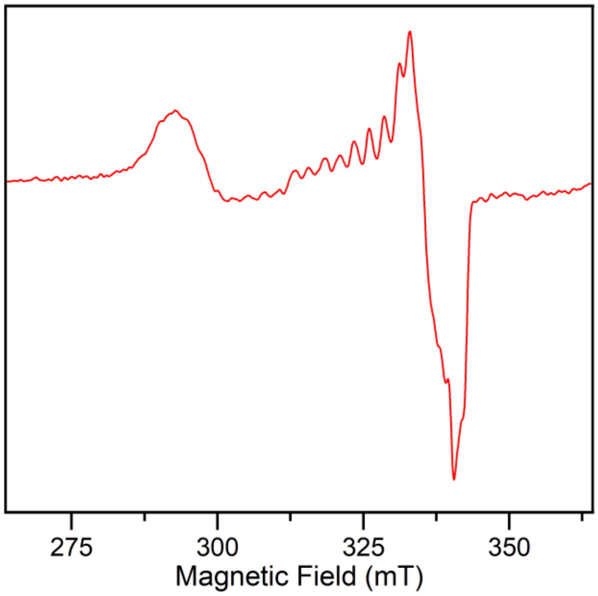

Figure S25. EPR spectrum of $Co(acac)_2$(dppBz) in a frozen toluene solution. The EPR spectrum was collected at 77 K using 1 mW microwave power,operating at 9.43 GHz. The EPR spectra is axial with g = [2.31, 2.02, 2.00]. Hyperfine interactions from ^{59}Co (I=7/2, 100%) resulted in additional splitting of the high field signal with an estimated hyperfine constant of 25 Gauss.
